# Novel α‐amino‐3‐hydroxy‐5‐methyl‐4‐isoxazole‐propionic acid receptor (AMPAR) potentiator LT‐102: A promising therapeutic agent for treating cognitive impairment associated with schizophrenia

**DOI:** 10.1111/cns.14713

**Published:** 2024-04-14

**Authors:** Xueyu Qi, Xueli Yu, Long Wei, Han Jiang, Jiangwen Dong, Hongxing Li, Yingying Wei, Liansheng Zhao, Wei Deng, Wanjun Guo, Xun Hu, Tao Li

**Affiliations:** ^1^ Affiliated Mental Health Center & Hangzhou Seventh People's Hospital and School of Brain Science and Brain Medicine Zhejiang University School of Medicine Hangzhou China; ^2^ Liangzhu Laboratory, MOE Frontier Science Center for Brain Science and Brain‐Machine Integration, State Key Laboratory of Brain‐Machine Intelligence Zhejiang University Hangzhou China; ^3^ NHC and CAMS Key Laboratory of Medical Neurobiology Zhejiang University Hangzhou China; ^4^ The Psychiatric Laboratory, the State Key Laboratory of Biotherapy West China Hospital of Sichuan University Chengdu Sichuan China; ^5^ The Clinical Research Center and Department of Pathology, The Second Affiliated Hospital Zhejiang University School of Medicine Zhejiang Hangzhou China

**Keywords:** alpha‐Amino‐3‐hydroxy‐5‐methyl‐4‐isoxazolepropionic acid receptor, brain‐derived neurotrophic factor, cognitive function, potentiator, schizophrenia

## Abstract

**Aims:**

We aimed to evaluate the potential of a novel selective α‐amino‐3‐hydroxy‐5‐methyl‐4‐isoxazole‐propionic acid receptor (AMPAR) potentiator, LT‐102, in treating cognitive impairments associated with schizophrenia (CIAS) and elucidating its mechanism of action.

**Methods:**

The activity of LT‐102 was examined by Ca^2+^ influx assays and patch‐clamp in rat primary hippocampal neurons. The structure of the complex was determined by X‐ray crystallography. The selectivity of LT‐102 was evaluated by hERG tail current recording and kinase‐inhibition assays. The electrophysiological characterization of LT‐102 was characterized by patch‐clamp recording in mouse hippocampal slices. The expression and phosphorylation levels of proteins were examined by Western blotting. Cognitive function was assessed using the Morris water maze and novel object recognition tests.

**Results:**

LT‐102 is a novel and selective AMPAR potentiator with little agonistic effect, which binds to the allosteric site formed by the intradimer interface of AMPAR's GluA2 subunit. Treatment with LT‐102 facilitated long‐term potentiation in mouse hippocampal slices and reversed cognitive deficits in a phencyclidine‐induced mouse model. Additionally, LT‐102 treatment increased the protein level of brain‐derived neurotrophic factor and the phosphorylation of GluA1 in primary neurons and hippocampal tissues.

**Conclusion:**

We conclude that LT‐102 ameliorates cognitive impairments in a phencyclidine‐induced model of schizophrenia by enhancing synaptic function, which could make it a potential therapeutic candidate for CIAS.

## Graphical abstract

The challenge of improving cognitive impairment associated with schizophrenia (CIAS) persists due to the limited availability of novel treatment options and drugs. Our study introduces a novel AMPAR potentiator, LT‐102, which could be a potential therapeutic candidate for CIAS by modifying synaptic plasticity and glutamate signaling.

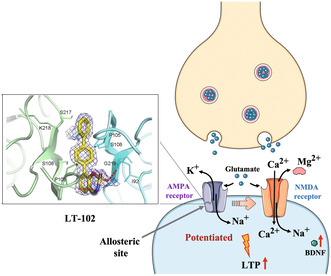

## 1 INTRODUCTION

Schizophrenia is a pervasive, severe, and chronic mental disorder characterized by positive (e.g., hallucinations and delusions), negative (e.g., avolition and self‐neglect), and cognitive symptoms (e.g., impaired attention and memory).^1^ While antipsychotic drugs targeting D2 dopamine receptors effectively manage positive symptoms, their impact on cognitive impairment associated with schizophrenia (CIAS) is less pronounced.^2^, ^3^ As patients and caregivers frequently report CIAS as one of the most burdensome aspects of the condition,^4^ it underlines the urgent need for novel therapeutic strategies.

The pathophysiology of schizophrenia, although not fully understood, is believed to stem from complex gene–environment interactions. Compelling evidence suggests that synaptic hypofunction of glutamatergic signaling at N‐methyl‐d‐aspartate receptors (NMDARs) contributes to the cognitive impairment seen in schizophrenia.^5^, ^6^ Indeed, blocking NMDARs pharmacologically triggers behavioral changes mimicking schizophrenia symptoms in healthy individuals and experimental animals.^7^, ^8^ Moreover, administration of the NMDAR antagonist ketamine exacerbates cognitive dysfunction in patients with schizophrenia.^9^ Consequently, restoring NMDAR function presents a potentially promising approach for CIAS treatment. Encouragingly, recent decades have witnessed significant advances in the development of NMDAR‐positive allosteric modulators, many of which have proven effective in alleviating some behavioral and cognitive symptoms in animal models of schizophrenia.^10^ However, a narrow therapeutic window exists for these NMDAR modulators, as there is a delicate balance between their beneficial and harmful effects, with overactivation leading to neurotoxicity and augmented neuronal death.^11^, ^12^, ^13^

Α‐amino‐3‐hydroxy‐5‐methyl‐4‐isoxazole‐propionic acid receptors (AMPARs), a type of ionotropic glutamate receptors, are integral for maintaining glutamate homeostasis and cognitive function.^14^, ^15^ AMPAR‐related functional defects have been implicated in the pathophysiology of schizophrenia. Emerging research suggests that regulating altered glutamate neurotransmission via activation of AMPARs could be a promising strategy for CIAS treatment. AMPARs mediate the majority of rapid excitatory transmission in the brain and play a crucial role in synaptic plasticity modulation. Long‐term potentiation (LTP), a form of synaptic plasticity involved in learning and memory formation, relies on the synaptic incorporation of AMPARs.^16^ Moreover, AMPAR and NMDAR function in a coupled and interdependent manner. AMPARs are crucial for restoring NMDAR‐mediated signal transduction and function. Activation of AMPARs initiates depolarization, leading to the removal of the channel‐blocking magnesium ions of NMDARs, thus overcoming their blockade.^17^ Furthermore, AMPARs have a lower affinity for glutamate and dissociate more rapidly than NMDARs, making them less likely to induce glutamate neurotoxicity.^18^, ^19^ These characteristics make AMPARs potential therapeutic targets for the treatment of CIAS.

However, due to the risk of receptor desensitization and excitotoxicity associated with agonist‐induced AMPAR activation,^20^, ^21^ AMPAR potentiators are considered more attractive for their selectivity and safer profiles. Despite promising early research, few potentiators have advanced to early clinical development stages due to challenges such as low potency and narrow therapeutic windows.^22^, ^23^, ^24^ Therefore, there is a pressing need for novel AMPAR potentiators with higher potency and reduced agonistic effects, aiming to address the treatment of psychiatric and neurological diseases.

A recent study demonstrated that the AMPAR potentiator TAK‐137, which exhibited a lower seizure risk compared to the comparator compound LY451646 in animal (rats and monkeys) models, is a promising candidate with potent pro‐cognitive effects.^25^ Further to this concept, our study introduces a novel compound, LT‐102, an AMPAR potentiator demonstrating greater potency than its counterpart, TAK‐137. Remarkably, the intraperitoneal administration of LT‐102 at a very low dose (0.01 or 0.05 mg/kg) successfully improved cognitive deficits in a mouse model of schizophrenia induced by phencyclidine. These findings position LT‐102 as a promising lead compound for the treatment of cognition‐related neuropsychiatric disorders such as CIAS.

## 2 MATERIALS AND METHODS

### 2.1 Chemicals

Phencyclidine (PCP) was generously gifted by the Changchun Institute of Applied Chemistry, Chinese Academy of Science. LT‐102 was synthesized in our laboratory, as outlined in our previous publication.^26^ TAK‐137 was prepared according to methods previously reported (patent WO2012020848). All other materials were procured from standard commercial sources.

### 2.2 Animals

We sourced specific pathogen‐free (SPF) embryonic day 18 (E18) pregnant Sprague Dawley rats, and male C57BL/6J mice weighing 20–22 g (6–7 weeks old) from the Animal Laboratory Center of Sichuan University (Chengdu, China). Mice were accommodated in clear polypropylene cages, with five animals per cage, on corn cob bedding. These conditions were environmentally controlled at 22°C with a 12‐h dark/light cycle and a relative humidity of 60%. All animals had free access to standard laboratory chow and water ad libitum. All experimental procedures were performed in accordance with the Guide for the Care and Use of Laboratory Animals (National Institutes of Health). The study protocols adhere to the ARRIVE guidelines and received approval from the Institutional Animal Care and Use Committee of Sichuan University (Approval No. 2020135A).

### 2.3 Primary culture of hippocampal neurons

Primary hippocampal neurons were prepared as described earlier.^24^ Briefly, the hippocampi of rat embryos were isolated from Sprague Dawley rats on embryonic day 18 in ice‐cold Hanks' Balanced Salt Solution and dispersed into single cells. Dissociated cells were plated on poly‐D‐lysine‐coated 96‐well plates (Corning, New York) at a density of 5 × 10^4^ cells/well for a Ca^2+^ influx assay, 1.5 × 10^4^ cells/well in a 35‐mm dish for whole‐cell voltage‐clamp recordings, and 3 × 10^5^ cells per well in 12‐well plates (Corning, USA) for western blot analysis. Cells were then cultured in Neurobasal Medium (Thermo Fisher Scientific, USA) supplemented with 1% B27 (Thermo Fisher Scientific, USA), 2 mM L‐glutamine (Thermo Fisher Scientific, USA), 100 U/mL penicillin, and 100 μg/mL streptomycin (Sigma‐Aldrich, USA). These cells were incubated for 5 days at 37°C in 5% CO_2_ for the Ca^2+^ influx assay and western blot analysis.

### 2.4 Ca^2+^ influx assay using primary neurons

The Ca^2+^ influx assay was carried out as recommended by the manufacturer (Dojindo, Japan), in line with previously described procedures.^24^ Briefly, cultured neurons were incubated with Fluo‐4 AM fluorescent calcium indicator dye solution with 1.25 mM Probenecid (TCI, Japan) for 60 min at 37°C in 5% CO_2_. This was followed by two washes with D‐Hanks Balanced Salt Solution (Ca^2+^ free) to remove the extracellular Fluo‐4 AM. The relative intracellular Ca^2+^ levels triggered by a compound were monitored using a fluorometric imaging plate reader (Molecular Devices SpectraMax i3, USA). The activity of each compound was defined as the fluorescence intensity integrated over the total measurement period, with 0% activity defined in the presence of only 0.1% DMSO and 100% activity in the presence of 5 μM s‐AMPA and 10 μM TAK‐137.

### 2.5 Western blot analysis

Following five days in culture, neuronal cells were exposed to compounds with or without s‐AMPA (1 μM) and further incubated at 37°C for 24 h in a 5% CO_2_ humidified incubator. Subsequently, total protein was harvested using Cell Lysis Buffer (Invent Biotechnologies; SD‐001) in compliance with the manufacturer's guidelines. Electrophoresis was performed using AnyKD‐PAGE gel (ATGene, Chongqing, China, Cat# ATG0046). Polyvinylidene fluoride membranes with transferred proteins were blocked with Protein Free Rapid Blocking Buffer (EpiZyme, Shanghai, Cat# PS108) for 15 min and left overnight at 4°C with primary antibodies: phospho‐GluA1 (at Ser831, Cell Signaling Technology, USA, Cat# 75574S, 1:1000), GluA1 (Cell Signaling Technology, USA, Cat# 2983, 1:1000), brain‐derived neurotrophic factor (BDNF; Abcam, UK, Cat# ab108319, 1:1000), and Beta‐Actin (Proteintech, USA, Cat# 66009, 1:10,000), Membranes were incubated the next day with horseradish peroxidase‐linked secondary antibodies (Cell Signaling Technology, Danvers, USA; polyclonal anti‐rabbit IgG, Cat# 7074 or polyclonal anti‐mouse IgG, Cat# 7076, 1:2000) for 1 h at room temperature and then exposed to Clarity Western ECL Substrate (Cat #1705061; Bio‐Rad, USA) or Super ECL Detection Reagent (Yeasen, Shanghai, China, Cat# 36208ES76) with a ChemiDoc MP system (Bio‐Rad, USA). Optical densities were analyzed using ImageJ software (Bethesda, Maryland, USA).

### 2.6 AMPA‐evoked currents in rat primary hippocampal neurons

After 14 days of culture, we employed whole‐cell voltage‐clamp recordings on hippocampal neurons. We used patch electrodes with a tip resistance of 3–5 MΩ, filled with an intracellular solution. This solution was composed of 5 mM NaCl, 140 mM K‐Gluconate, 1 mM MgCl_2_•6H_2_O, 0.1 mM CaCl_2_•6H_2_O, 1 mM EGTA, 10 mM HEPES, and 2 mM Mg‐ATP and was adjusted to pH 7.2 with KOH. The extracellular solution contained 140 mM NaCl, 3.5 mM KCl, 1 mM MgCl_2_•6H_2_O, 2 mM CaCl_2_•2H_2_O, 10 mM D‐glucose, 10 mM HEPES, and 1.25 mM NaH_2_PO_4_•2H_2_O and was adjusted to pH 7.4 with NaOH. We maintained the neurons at a voltage clamp of 80 mV. Steady‐state inward currents were prompted via the application of specific agonists and compounds through a carefully controlled perfusion system. We collected and evaluated the experimental data using the pClamp 10 software (Axon Instruments, Foster City, CA, USA). Current magnitudes were normalized to the currents stimulated by 10 μM s‐AMPA.

### 2.7 hERG tail current in HEK293 cells

To record hERG tail currents, a patch‐clamp assay was performed using a QPatch 48X automated electrophysiology platform (Sophion Bioscience, Ballerup, Denmark).^27^ The extracellular solution was the same as the one used in section 2.6. The intracellular solution employed consisted of 20 mM KCl, 115 mM K‐aspartate, 1 mM MgCl_2_•6H_2_O, 5 mM EGTA, 10 mM HEPES, and 2 mM Na_2_‐ATP. The pH of the intracellular solution was adjusted to 7.2 using KOH.

### 2.8 Kinase‐inhibition assays

The in vitro kinase inhibition potency of LT‐102 against a panel of 310 kinases was measured at 10 μmol/L, using the Homogeneous Time‐Resolved Fluorescence (HTRF) method. This service was commercially provided by ICE Bioscience Co., Ltd., China. Kinase inhibition assays were performed based on previously described methods.^28^ In brief, all reagents and samples were prepared, which included kinase substrate solution, ATP solution, kinase enzyme solution, and LT‐102, into a 384‐well microplate. After incubating this setup at 25°C for 1 h, allowing the kinase reaction to transpire, we terminated the reaction with a stop solution. We then added HTRF detection reagents, which include two specific antibodies marked with donor and acceptor fluorophores, into the wells. Another round of incubation allowed for the development of the HTRF signal. The fluorescence signal was subsequently measured using a compatible HTRF plate reader (BMG Labtech). The signal was expressed as the ratio of acceptor fluorescence (measured at 665 nm) to donor fluorescence (measured at 620 nm) and multiplied by 10,000 to enhance sensitivity.

### 2.9 Preparation of hippocampal slices

Mice were deeply anesthetized using isoflurane and subsequently decapitated. Mouse hippocampal slices (300 μm) were prepared using a vibratome (Leica, VT 1000 S, Germany). The slicing procedure was performed in ice‐cold artificial cerebrospinal fluid that was oxygenated to maintain optimal conditions. The ACSF composition consisted of 125 mM NaCl, 2.5 mM KCl, 1.25 mM NaH_2_PO_4_, 25 mM NaHCO_3_, 25 mM D‐Glucose, 2 mM CaCl_2_, and 1.5 mM MgCl_2_. The ACSF solution was continuously bubbled with a mixture of 95% O_2_ and 5% CO_2_ to maintain a pH of 7.4. After slicing, the hippocampal slices were transferred to oxygenated ACSF at room temperature and recovered for 60 min before initiating further experiments.

### 2.10 LTP recordings

The protocol for recording field excitatory postsynaptic potentials (fEPSPs) was described previously.^29^ Field recordings were conducted in the CA1 region of the hippocampus, and the Schaffer collateral fibers were stimulated using a bipolar electrode. To induce long‐term potentiation (LTP), a theta‐burst stimulation (TBS) protocol was employed. This protocol consisted of five trains of bursts, each containing four pulses at a frequency of 100 Hz. The stimulation was repeated four times with intervals of 10 s between each train. The magnitude of the LTP was calculated by comparing the average slopes of the fEPSPs during the last 10 min of a recording with the baseline slopes recorded before stimulation. Data acquisition and analysis were performed using pClamp 11 software (Axon Instruments, Foster City, CA, USA).

### 2.11 Miniature excitatory postsynaptic current recordings

To investigate miniature excitatory postsynaptic currents (mEPSCs), whole‐cell patch‐clamp recordings were conducted on mouse hippocampal slices following a previously established protocol.^30^ The recordings were performed specifically on CA1 pyramidal neurons. The components of the intracellular solution included the following: 140 mM K‐gluconate, 2 mM MgCl_2_, 10 mM HEPES, 8 mM KCl, 2 mM Na_2_‐ATP, and 0.2 mM Na_2_‐GTP. The pH of the solution was adjusted to 7.2 using KOH. The extracellular solution, which bathed the hippocampal slices, consisted of 125 mM NaCl, 2.5 mM KCl, 1.25 mM NaH_2_PO_4_•2H_2_O, 25 mM NaHCO_3_, 10 mM D‐Glucose, 2 mM CaCl_2_•2H_2_O, and 1.5 mM MgSO_4_. This solution was equilibrated with a mixture of 95% O_2_ and 5% CO_2_ for at least 30 min, until its pH was 7.4. The resistance of the pipettes was 4–6 MΩ. Additionally, the extracellular solution contained antagonists: bicuculline (10 μM, Sigma‐Aldrich, USA) to block GABA_A_ receptors, 2‐amino‐5‐phosphonopentanoic acid (20 μM, Sigma‐Aldrich, USA) to block NMDA receptors, and tetrodotoxin (TTX, 1 μM, Sigma‐Aldrich, USA) to block action potentials. During the mEPSC recordings, CA1 pyramidal neurons were voltage‐clamped at −70 mV without synaptic stimulation. The recordings were performed using pClamp11 software. Baseline recordings were obtained for 10 min before the administration of LT‐102. Following LT‐102 administration, recordings were performed for an additional 10 min.

### 2.12 AMPAR‐mediated excitatory synaptic current recordings

The resistance of pipettes and the intracellular and the extracellular solution were the same as in the section 2.11, with the exception that TTX was not added to the extracellular solution. The AMPAR‐mediated EPSC amplitude was measured as the peak EPSC amplitude at a holding potential of −70 mV. The paired‐pulse ratio (PPR) was calculated as the ratio of the amplitude of the second response to the first at each interpulse interval (20, 50, 100, and 200 ms).

### 2.13 Drug administration

PCP was prepared by dissolving it in normal saline, and the solution was freshly prepared daily. LT‐102 or normal saline was dissolved in a solution containing 0.1% DMSO and 0.4% Tween 80. All treatments were administered intraperitoneally (i.p.) at 10 mL/kg. The dosing regimen followed an established protocol.^31^ Forty mice, each averaging 8 weeks of age, were randomly assigned to four groups, with ten mice per group. To ensure consistency in behavioral outcomes, mice were selected from the same batch to mitigate bias from repeated testing. Age and weight matching was conducted, and random group allocation was performed prior to the experiment to bolster result reliability. The animals in the vehicle‐treated group received saline (0.9% NaCl), while the PCP treatment group received PCP at 10 mg/kg twice daily for seven consecutive days. Following a 7‐day wash‐out period, the treatment group received a single dose of either 0.01 mg/kg or 0.05 mg/kg LT‐102, while the control and PCP model groups received saline injection daily for 7 days. Behavioral testing was initiated at the conclusion of drug treatment.

### 2.14 Behavioral tests

#### 2.14.1 Morris water maze test

The Morris water maze test was conducted following a previous protocol^32^ with minor modifications. The mice were trained for five consecutive days after the last administration of LT‐102 or the vehicle. During each training day, the mice underwent three trials, one per quadrant, to locate a fixed platform (10 cm in diameter) positioned 1 cm below the water surface. The surrounding walls of the maze were marked with different geometric shapes as visual cues. In each training trial, the mouse was placed randomly in the water at one of three starting points and given 60 s to find the hidden platform. If the mouse failed to find the platform within the time limit, it was gently guided to the platform by the experimenter. On the fifth day, a probe trial was conducted. The mouse was released from the quadrant opposite platform and allowed to swim for 60 s. Throughout all trials, the swimming paths of the mice were recorded using the Noldus Media Recorder software (Noldus Information Technology, Netherlands). The recorded videos were analyzed using EthoVision XT video tracking software (Noldus Information Technology, Netherlands), which traced the locations of the animals and provided data for further analysis.

#### 2.14.2 Novel object recognition test

Novel object recognition tests were conducted as a previously described protocol.^33^ The mice were allowed to habituate in a test chamber measuring 50 × 50 × 40 cm. On day 1, the mice were placed in a conditioning box for 30 min and returned to their home cages. On day 2, during the acquisition trial, the mice were given 10 min to explore two identical objects labeled A1 and A2. On day 3, during the retention trial, the mice were presented with a familiar object (the same as A1 or A2) from the acquisition trial and a novel object. They were allowed to explore the objects for 10 min. During the trials, the exploration time for each object was traced and analyzed using EthoVision XT video tracking software (Noldus Information Technology, Netherlands). Exploration was defined as licking, sniffing, or touching an object. While actions such as leaning against the object to look upward or standing or sitting on the object were excluded from the analysis. Two measures were calculated based on the exploration times. First, the exploration preference was calculated as time spent exploring one object divided by the total exploration time and multiplied by 100 (%). Second, the novel object recognition index was calculated as the time spent exploring the novel object divided by the total interaction time and multiplied by 100 (%).

### 2.15 Crystallization of hGluA2 with compounds

DNA encoding residues 413–795 of human GluA2 (hGluA2), with amino acids 528–652 replaced by the amino acid GT, was synthesized by Shanghai Sangon Biotech Inc. The synthesized DNA was then cloned into a PET28a expression vector containing an N‐terminal His‐MBP tag followed by a TEV protease site. The expression vector was introduced into *Escherichia coli* BL21(DE3) competent cells. The transformed cells were allowed to undergo overnight expression at 16°C. After the expression period, the cells were harvested and disrupted by sonication in a lysis buffer containing 20 mM Tris–HCl, 250 mM NaCl, and pH 8.0. The cell lysate was centrifuged at 12,000 × *g* for 30 min at 4°C. The protein of interest, tagged with the His‐MBP fusion, was purified using NTA affinity beads (GE Healthcare, Little Chalfont, UK). Subsequently, TEV protease treatment was employed to remove the N‐terminal His‐MBP tag. To exclude the TEV protease and the released His‐MBP tag, additional NTA affinity beads (GE Healthcare) and MBP resin (GE Healthcare) were utilized. Finally, the tag‐free hGluA2 was purified through size‐exclusion chromatography utilizing a Superdex 200 column (GE Healthcare). The final buffer for this purification step contained 10 mM HEPES, pH 7.0, 20 mM NaCl, and 1 mM EDTA.

The purified hGluA2 protein was concentrated at 15 mg/mL. It was then incubated with compound LT‐102 at a molar ratio of 1:5 on ice for 0.5 h to allow binding. Crystals of the hGluA2/LT‐102 complex were obtained using the vapor diffusion hanging drop method, with the reservoir buffer consisting of 22% (w/v) PEG8000, 0.1 M NaAC at pH 5.0, and 0.2 M (NH_4_)_2_SO_4_. A cryoprotectant solution containing 20% glycerol was added to the reservoir solution. The crystals were then flash‐frozen in liquid nitrogen. Diffraction data for the hGluA2/LT‐102 complex were collected at the beamline BL19U1 of the Shanghai Synchrotron Radiation Facility at 100 K. The collected data were processed using XDS software to obtain the diffraction intensities. To determine the structure of the hGluA2/LT‐102 complex, a molecular replacement was performed using Phaser. A previously reported structure with the PDB code 2XHD was used as the search model.^34^ Model building was carried out using Coot, and the complex structure was refined using PHENIX.

### 2.16 Statistical analysis

All data were analyzed using Prism 8 (GraphPad Software Inc., San Diego, CA). The results were expressed as the mean ± standard error of the mean (SEM). Nonlinear regression analyses were performed to generate dose–response curves and calculate EC_50_ values. They evaluated the normality of the data distribution using the Shapiro–Wilk test. To determine differences among groups, we employed either one‐way or two‐way ANOVA with Tukey's post hoc test for parametric data, or Kruskal‐Wallis test for nonparametric data, followed by Dunn's post hoc test. Student's *t*‐test was used to compare differences between two groups. Statistical significance was defined as a *p*‐value less than 0.05 (*p* < 0.05).

## 3 RESULTS

### 3.1 LT‐102 is an AMPAR potentiator with little agonistic effect

**FIGURE 1:**
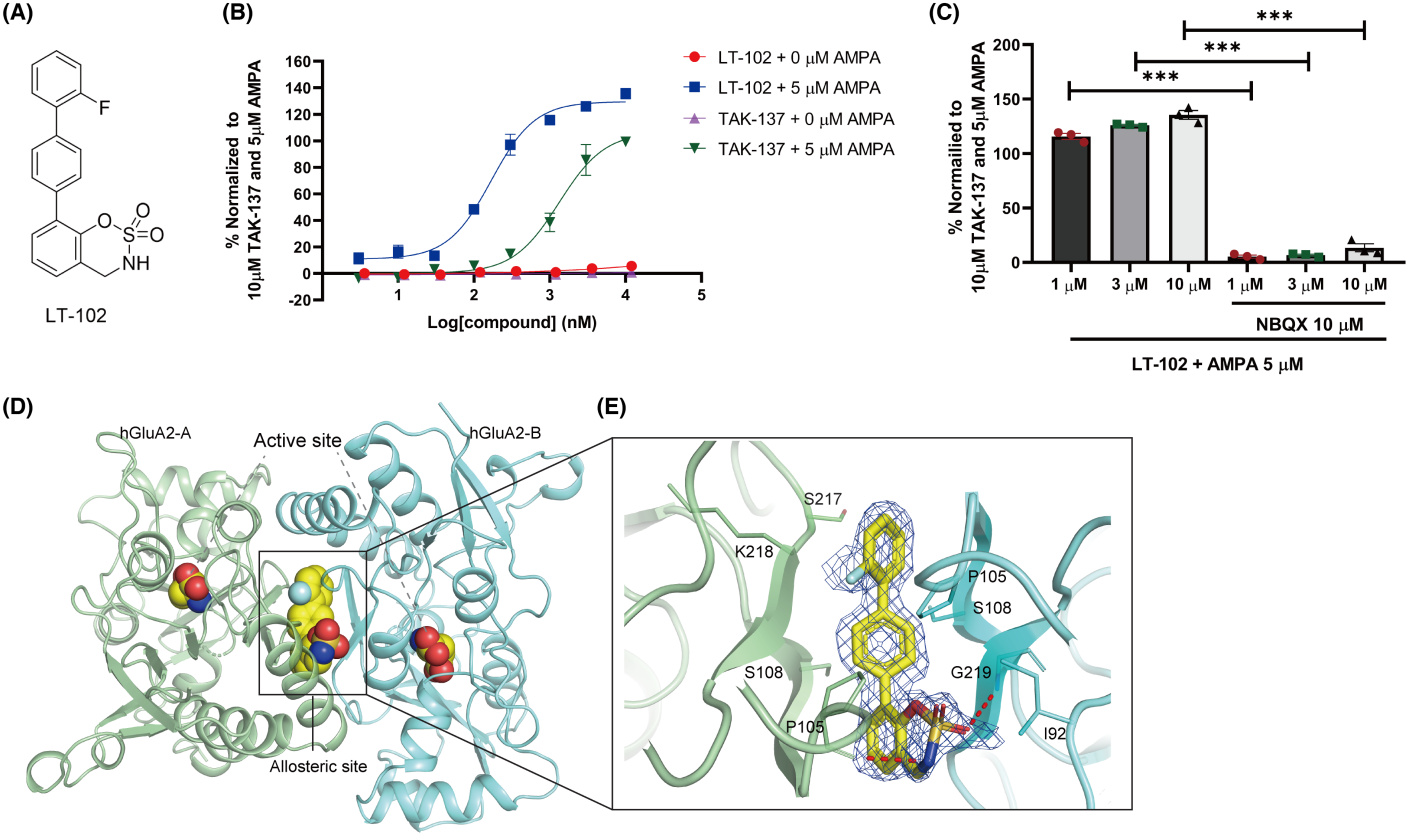
Effects of LT‐102 treatment on primary rat hippocampal neurons and the co‐crystal structure of hGluA2 with LT‐102. (A) Chemical structure of LT‐102. (B) Concentration‐dependent effect of LT‐102 and TAK‐137 treatments on Ca^2+^ influx, both in the presence or absence of 5 μM s‐AMPA (*n* = 3). (C) Effects of LT‐102 treatment, combined with 5 μM s‐AMPA, on Ca^2+^ influx in the presence or absence of 10 μM NBQX (*n* = 3, Student's *t*‐test). (D) The overall structure of the hGluA2/LT‐102 complex. (E) 3D‐detailed interactions between LT‐102 and hGluA2; hydrogen bonds are illustrated with red dashes. Data are shown as the mean ± SEM, with ***p < 0.001.

To identify novel AMPAR potentiators with reduced agonistic effects, we performed compound screening using a self‐designed chemical library, using TAK‐137 as a reference compound. Through a Ca^2+^ influx assay performed on primary rat hippocampal neurons, we identified a novel AMPAR potentiator called LT‐102 (Figure 1A).^26^ TAK‐137 has previously been reported as an efficient and selective AMPAR potentiate with minimal agonistic effect on primary neurons.^25^ Therefore, we compared the effects of TAK‐137 and LT‐102 using a Ca^2+^ influx assay. The EC_50_ and E_max_ values of LT‐102 in the presence of an agonist s‐AMPA (5 μM) were determined to be 0.169 μM and 129.6%, respectively, while those of the positive control TAK‐137 were 1.323 μM and 106.8%, respectively (Figure 1B). Similar to TAK‐137, LT‐102 exhibited minimal agonistic effect in the absence of s‐AMPA (Figure 1B). Furthermore, pretreatment with the competitive AMPAR antagonist NBQX significantly suppressed the agonist‐dependent activity of LT‐102, indicating that LT‐102 acted on AMPARs (Figure 1C). X‐ray crystallography was employed to investigate further the binging interactions of LT‐102 with the ligand‐binding domain (LBD) of the AMPAR subunit GluA2. X‐ray data collection and refinement statistics are listed in Table S1. The co‐crystal structure revealed that the LBD of GluA2 adopts a classic LBD fold and that LT‐102 binds to a hydrophobic pocket formed by LBD residues, similar to other AMPAR potentiators (Figure 1D). LT‐102 was observed to bind to allosteric sites on the LBD, forming two hydrogen bonds with residues Gly 219 of S1 and Pro 105 of S2 in the LBD (Figure 1E). The two hydrogen bonds between LT‐102 and the LBD likely contributed to the high potentiation of LT‐102, leading to increased AMPAR activity. These findings demonstrate that LT‐102 is an AMPAR potentiator with minimal agonistic effect and exhibits higher potency and efficacy.

### 3.2 LT‐102 selectively potentiates AMPA‐evoked currents in primary rat hippocampal neurons

**FIGURE 2:**
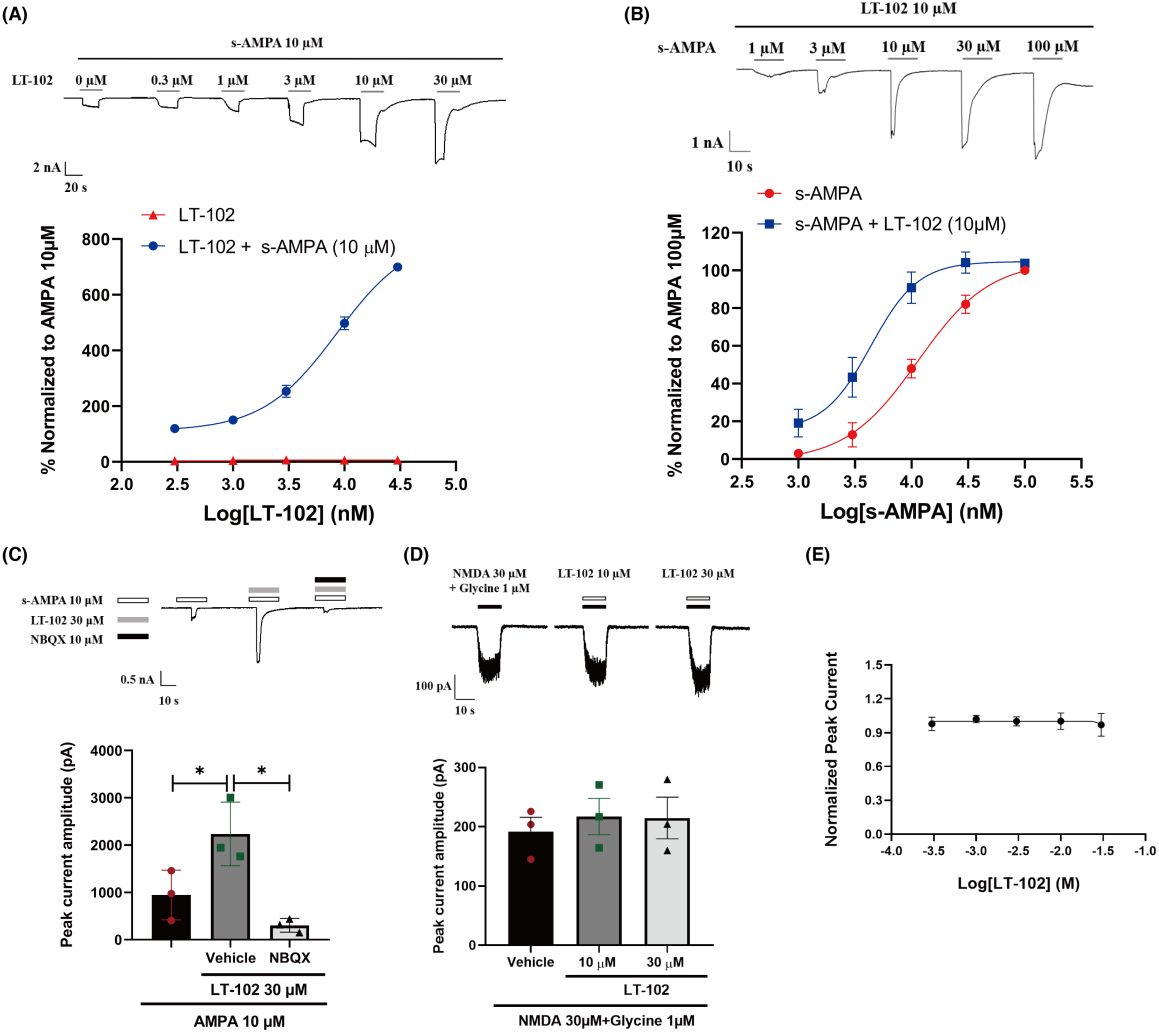
Selectivity of LT‐102. (A) Concentration‐dependent effects of LT‐102 treatment on AMPA‐evoked currents in the presence or absence of 10 μM s‐AMPA, normalized to the s‐AMPA (10 μM) response (*n* = 3). (B) Concentration‐dependent effects of s‐AMPA treatment on AMPA‐evoked currents in the presence or absence of 10 μM LT‐102, normalized to the s‐AMPA (100 μM) response (*n* = 3). (C) Effect of NBQX (10 μM) on AMPAR current (*n* = 3, Student's *t*‐test). (D) Effect of LT‐102 on NMDA‐activated currents (*n* = 3, one‐way ANOVA). (E) Dose–response relationship showing no inhibition of hERG by LT‐102 treatment (*n* = 3). *p < 0.05.

To further evaluate the efficacy and selectivity of LT‐102, we performed whole‐cell patch‐clamp recordings of primary rat hippocampal neurons. LT‐102 treatment significantly amplified AMPA‐evoked currents in a dose‐dependent manner in the presence of 10 μM s‐AMPA while exhibiting virtually no agonistic activity in the absence of s‐AMPA. The half‐maximal effective concentration (EC_50_) for LT‐102 was determined to be 8.558 μM, and the maximal response (E_max_) values represented an 813.8‐fold augmentation in the presence of 10 μM s‐AMPA (Figure 2A). Remarkably, LT‐102 at a concentration of 10 μM considerably amplified the potency of s‐AMPA, which led to a decline in its EC_50_ from 11.357 to 4.375 μM (Figure 2B). We further investigated the selectivity of LT‐102 and found that AMPA‐induced currents were almost entirely inhibited in the presence of 10 μM NBQX (Figure 2C). Given the often coupled and interdependent functions of AMPAR and NMDAR, we examined the effects of LT‐102 on NMDA‐mediated currents in the presence of NMDA (30 μM) and its co‐agonist glycine (1 μM). Delightfully, LT‐102 at 10 μM or 30 μM had no discernible enhancing or inhibiting effect on NMDA‐activated currents (Figure 2D). Additionally, patch‐clamp recordings demonstrated that LT‐102 treatment did not impact the hERG tail current in hERG‐HEK293 cells, implying a low risk of cardiotoxicity (IC_50_ > 30 μM, Figure 2E). To extend our understanding of LT‐102's selectivity, we examined its effects against a panel of 310 kinases. The results revealed that LT‐102 exerted less than 50% inhibitory activities against all tested kinases, indicating its high specificity (Figure S1; Table S2). These findings strongly suggest that LT‐102 is a highly selective AMPAR potentiator.

### 3.3 LT‐102 increased BDNF expression and GluA1 phosphorylation in an agonist‐dependent manner

**FIGURE 3:**
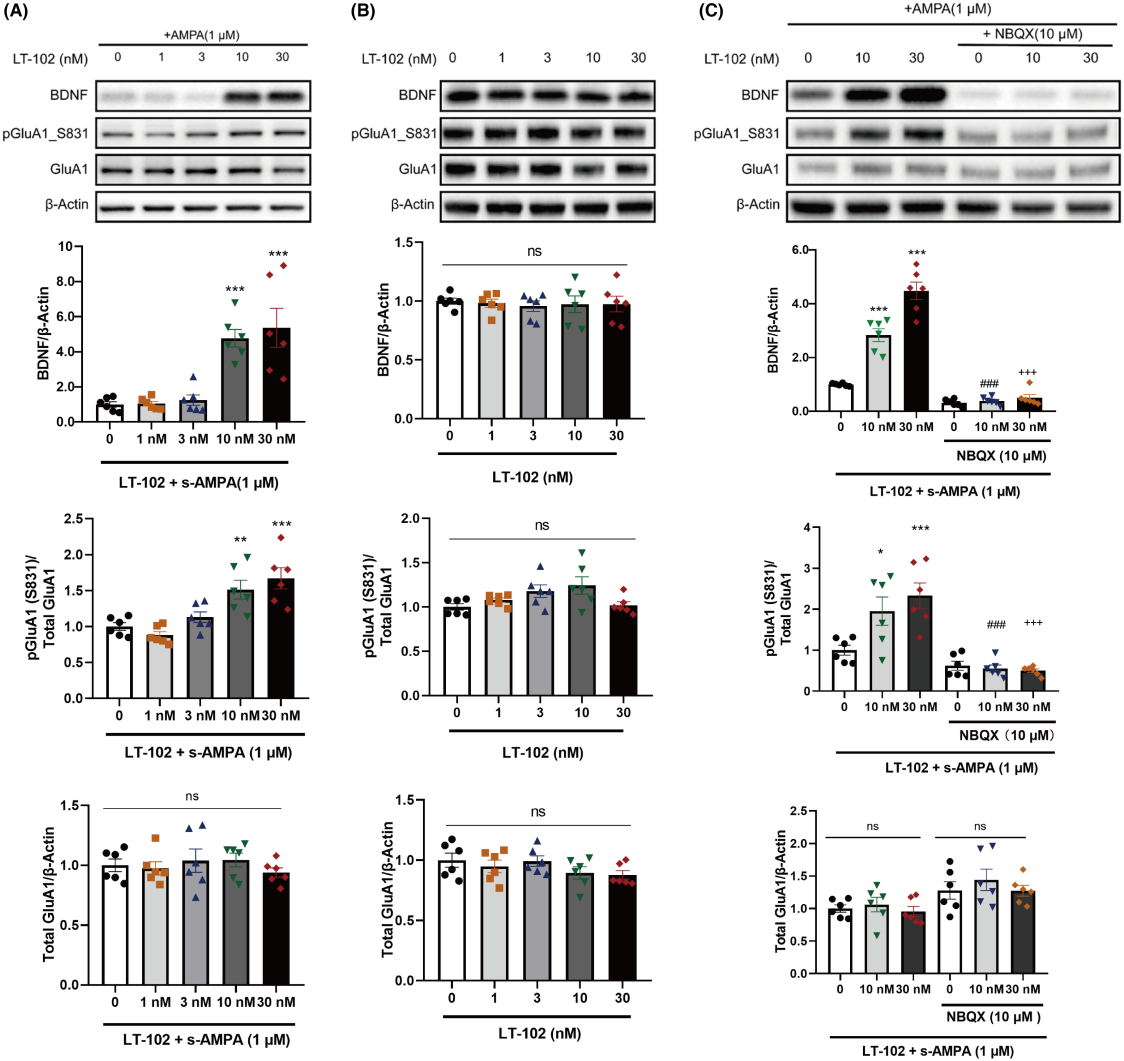
Effect of LT‐102 treatment on BDNF expression and GluA1 phosphorylation. (A) Expression of BDNF, the ratio of pGluA1/GluA1, and total GluA1 following 24 h of LT‐102 incubation in the presence of 1 μM s‐AMPA (*n* = 6, one‐way ANOVA). (B) Expression of BDNF, the ratio of pGluA1/GluA1, and total GluA1 following 24 h of LT‐102 incubation in the absence of s‐AMPA (*n* = 6, one‐way ANOVA). (C) Expression of BDNF, the ratio of pGluA1/GluA1, and total GluA1 were significantly inhibited by NBQX (10 μM) (*n* = 6, Two‐way ANOVA). *p < 0.05, **p < 0.01, ***p < 0.001 vs Control (0); ^###^ *p* < 0.001 vs LT‐102 (10 nM) + AMPA (1 μM); ^+++^ *p* < 0.001 vs LT‐102 (30 nM) + AMPA (1 μM); ns means no significance.

Given the crucial role of BDNF and GluA1 phosphorylation in synaptic plasticity modulation,^35^ we examined the impact of LT‐102 treatment on these factors in primary rat hippocampal neurons. The results revealed that LT‐102 treatment at 10 or 30 nM significantly enhanced BDNF protein expression and GluA1 phosphorylation in the presence of 1 μM s‐AMPA (Figure 3A). Conversely, no significant changes were observed in the absence of s‐AMPA (Figure 3B). Additionally, pretreatment with 10 μM NBQX effectively inhibited the LT‐102‐induced BDNF expression and GluA1 phosphorylation in the presence of 1 μM s‐AMPA (Figure 3C). These findings demonstrate that LT‐102 treatment promotes BDNF expression and GluA1 phosphorylation through the activation of AMPAR activation in an agonist‐dependent manner in vitro.

### 3.4 LT‐102 treatment enhanced long‐term potentiation (LTP) at CA1 hippocampal synapses

**FIGURE 4:**
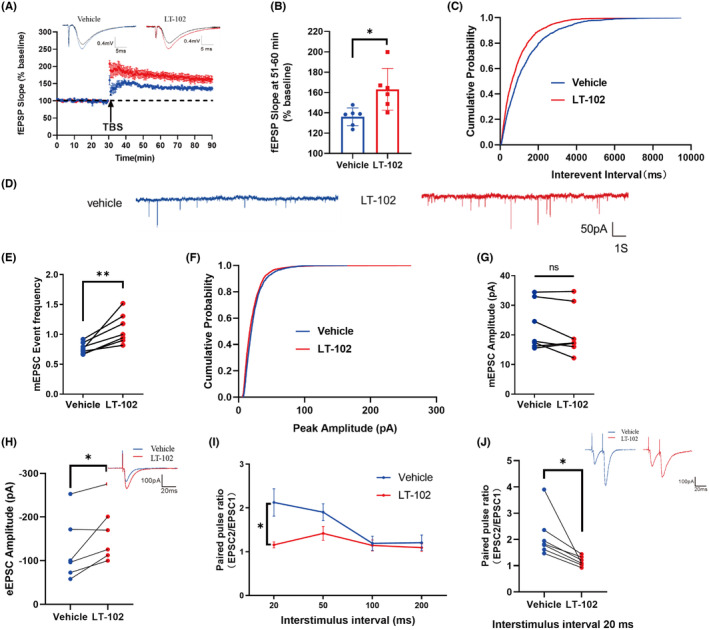
Electrophysiological assessment of LT‐102 treatment on mouse hippocampal slices. (A) Normalized mean fEPSP slope against time in the presence or absence of LT‐102 (*n* = 6). Inserts are representative signal averages of fEPSPs before (black) and after TBS in the presence (red) or absence (blue) of LT‐102. (B) Summary of the averaged fEPSP slope of the last 10 min (*n* = 6, Student's *t*‐test). (C) Cumulative distribution of inter‐event interval of mEPSCs before and during 10 μM LT‐102 treatment (*n* = 6). (D) Representative mEPSC signals were recorded at a holding potential of −70 mV in the presence or absence of LT‐102. (E) Histogram summarizing the frequency of mEPSCs between the two groups (*n* = 7, Student's *t*‐test). (F) Cumulative distribution of the amplitude of mEPSCs before and during 10 μM LT‐102 treatment (*n* = 6). (G) Histogram summarizing the amplitude of mEPSC between the two groups (*n* = 6, Student's *t*‐test). (H) Histogram summarizing the amplitude of AMPAR‐EPSCs between the two groups (*n* = 6, Student's *t*‐test). Inserts are representative traces of stimulus‐evoked EPSC before and during 10 μM LT‐102 treatment. (I) Summary diagram of the mean PPR measured at ISIs from 20 to 200 ms (*n* = 6). (J) Histogram summarizing the PPR at ISIs of 20 ms (*n* = 6, Student's *t*‐test). Inserts are representative voltage traces showing responses to pairs of stimuli (ISI: 20 ms). *p < 0.05, **p < 0.01, and ns means no significance.

To evaluate the effect of LT‐102 treatment on LTP in mouse hippocampal slices, field excitatory postsynaptic potentials (fEPSPs) were measured in CA1 pyramidal neurons through stimulation of the Schaffer collaterals. The results demonstrated a significant enhancement of the average fEPSP slope during the last 10 min following theta burst stimulation (TBS) compared to the vehicle control (Figure 4A,B). Additionally, the impact of LT‐102 treatment on synaptic transmission's intrinsic properties was investigated. The findings revealed a substantial increase in mEPSC frequency following LT‐102 administration (Figure 4C–E), while mEPSC amplitude remained unaffected (Figure 4F,G). Furthermore, the evoked EPSC (eEPSC) amplitude was significantly elevated (Figure 4H). To evaluate the potential contribution of presynaptic neurotransmitter release to the improvement of LTP induced by LT‐102, paired‐pulse facilitation (PPF) was assessed. PPF was significantly reduced from 2.13 ± 0.31 to 1.16 ± 0.07 at an interstimulus interval (ISI) of 20 ms (Figure 4I,J). Nevertheless, we found no significant difference at ISIs of 50–200 ms (Figure 4I). These data suggested that the facilitated effects of LT‐102 treatment on LTP may be attributed to an AMPAR‐mediated increase in presynaptic transmitter release.

### 3.5 LT‐102 treatment ameliorates PCP‐induced spatial memory deficits in mice

**FIGURE 5:**
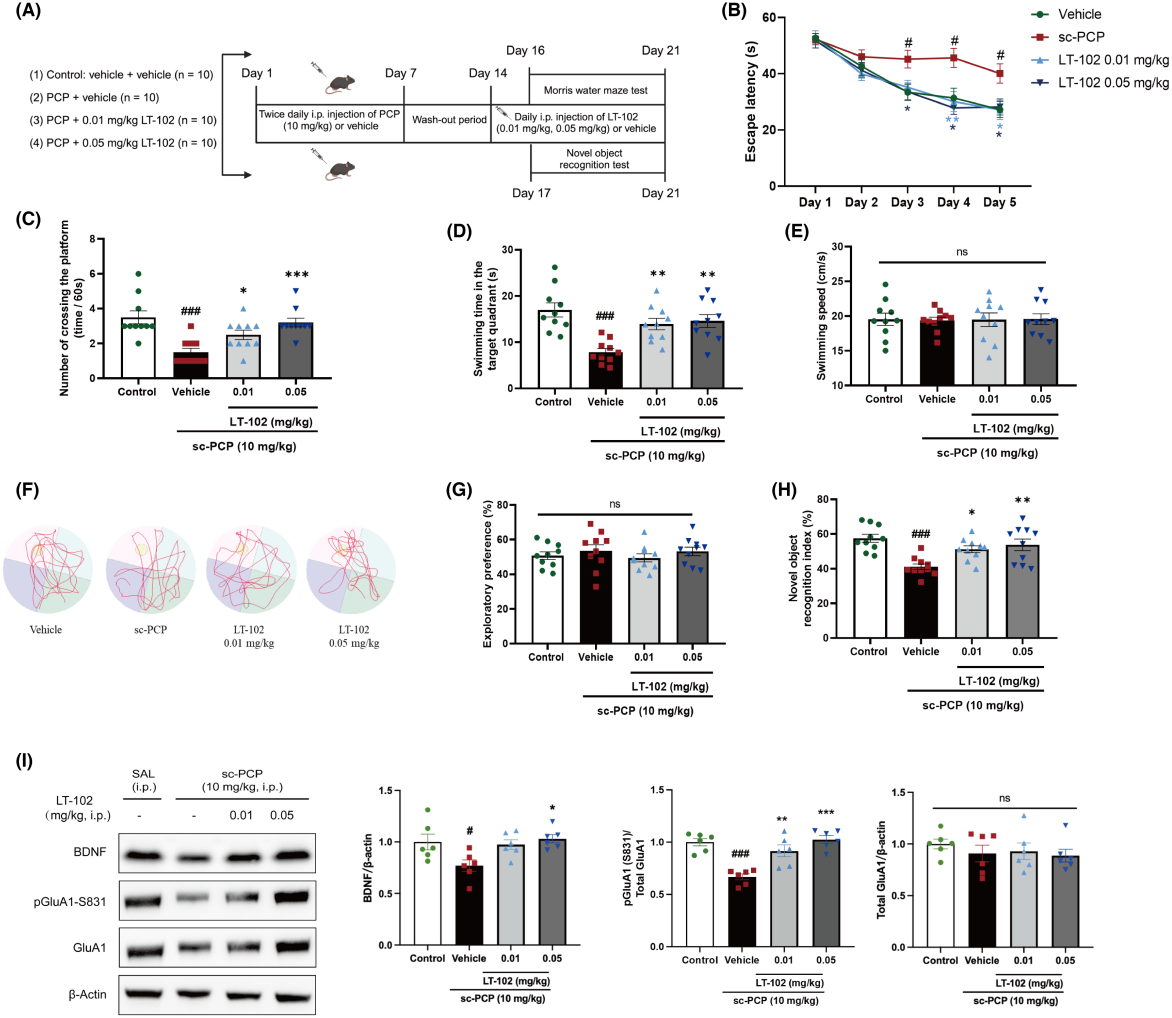
LT‐102 treatment ameliorates PCP‐induced learning and memory impairment in mice. (A) Scheme of the experimental procedure and timeline of the Morris water maze and novel object recognition tests. (B) The escape latency to find the platform during the training period. (*n* = 10, one‐way ANOVA). (C) Crossing times of the virtual platform during the probe test (*n* = 10, Kruskal‐Wallis). (D) The time in the target quadrant in the probe test (*n* = 10, one‐way ANOVA). (E) Swimming speeds of mice in the probe test (*n* = 10, one‐way ANOVA). (F) Representative trajectories of each group in the probe test. (G) Exploratory preference of the objects (*n* = 10, one‐way ANOVA). (H) Percentage of the novel object recognition index. (*n* = 10, one‐way ANOVA). (I) Expression of BDNF, GluA1, and the ratio of pGluA1/GluA1 in the hippocampus (*n* = 6, one‐way ANOVA). ^#^ *p* < 0.05, ^###^ *p* < 0.001 vs Control; *p < 0.05, **p < 0.01, ***p < 0.001 vs sc‐PCP group; ns means no significance.

To investigate the effect of LT‐102 treatment on schizophrenia‐associated spatial learning and memory dysfunction, we established a PCP‐induced mouse model based on previous protocols.^31^, ^36^ The Morris water maze and novel object recognition tests were used to gauge the efficacy of LT‐102 treatment. The dosing regimens are depicted in Figure 5A. Overall, the latencies to reach the platform decreased in all groups over the five training days in the Morris water maze (Figure 5B). Compared with the vehicle‐treated mice, PCP‐treated mice required significantly more time to reach the platform, and LT‐102‐treated mice found the platform faster from day 3 onwards (Figure 5B). In the probe test, the number of virtual platform crossings (Figure 5C) and time spent in the target quadrant (Figure 5D) were significantly lower for PCP‐treated mice compared to vehicle‐treated mice, indicating spatial learning and memory function deficits. This impairment was dose‐dependently mitigated by LT‐102 treatment (Figure 5C,D). Representative swimming traces from the probe trials are depicted in Figure 5F. Swimming speed did not differ significantly between the groups (Figure 5E). We further explored the effects of LT‐102 treatment on recognition memory using the novel object recognition test. During the habituation trial, we found no differences in exploratory preferences between the groups (Figure 5G). However, during the novelty recognition trial, PCP‐treated mice showed an apparent reduction in the discrimination index compared to the control, suggesting impaired recognition memory (Figure 5H). LT‐102‐treated mice demonstrated a significantly higher discrimination index in a dose‐dependent manner (Figure 5H). These results suggest that LT‐102 treatment dose‐dependently counteracts the spatial and recognition memory impairments observed in PCP‐treated mice. We also observed a significant decrease in the basal expression level of BDNF and the phosphorylation level of GluA1 in the hippocampus of PCP‐treated mice compared to the control group (Figure 5I). Interestingly, LT‐102 administration effectively reversed these reductions, suggesting that BDNF and the GluA1 subunit play crucial roles in mediating the treatment effect of LT‐102.

## 4 DISCUSSION

The alterations in AMPARs observed in genetic, transcriptomic, and proteomic studies of schizophrenia indicate enhancing AMPAR function is an intervention for improving CIAS,^37^, ^38^ In this study, we investigated the potential therapeutic efficacy of LT‐102, a recently developed novel AMPAR potentiator,^26^ for the treatment of CIAS. We employed both in vitro and in vivo assays to assess the effectiveness and selectivity of LT‐102.

There are several clinical studies underway investigating the therapeutic potential of AMPAR‐positive allosteric modulators (PAMs) across various phases of development. Firstly, a well‐documented AMPAR PAM, CX‐516 (Ampalex), has been studied for its potential cognitive‐enhancing effects. It has undergone clinical trials to assess its effectiveness in enhancing cognitive function in individuals with Alzheimer's disease and schizophrenia.^39^ Secondly, LY451395 has been studied for its potential benefits in treating depression and Alzheimer's disease.^40^, ^41^ Clinical trials have aimed to determine its efficacy and safety in patients with major depressive disorder. Thirdly, representing a novel approach within the AMPAR PAM category, JNJ‐40411813 has demonstrated potential in clinical studies for addressing neurological and psychiatric disorders.^42^ Ongoing clinical trials seek to establish its safety and effectiveness in diverse patient groups. Additionally, a range of novel AMPAR PAMs, including bivalent PAMs, are in the development stage, showing promise in preclinical evaluations for neuropsychiatric disorders.^43^ These studies highlight the active exploration and potential of AMPAR PAMs in addressing a spectrum of cognitive and mood disorders, underscoring the ongoing efforts to translate preclinical successes into clinical therapies. However, some AMPAR potentiators, such as LY451646, LY451395, and OXP1, have demonstrated a bell‐shaped response with agonistic effects, which can increase the risk of seizures.^24^, ^44^ More recently, TAK‐137 was discovered to have fewer agonistic effects and a wider safety margin against seizures.^25^ In our study, we demonstrated that LT‐102 exhibited higher efficacy than TAK‐137 in the Ca^2+^ influx assay with minimizing agonistic effect. Furthermore, we established the high selectivity of LT‐102 as an AMPAR potentiator. LT‐102 treatment potentiated AMPA‐evoked currents without affecting NMDA‐mediated currents or hERG tail currents in primary neurons. LT‐102 had only a weak inhibitory effect on all tested kinases. The favorable safety profile of LT‐102, with minimal agonistic effects and limited impact on other receptor systems, further strengthens its potential as a promising therapeutic agent for CIAS.

Numerous studies investigating schizophrenia have consistently shown an association between AMPARs and the underlying pathophysiology of schizophrenia. This association is supported by the crucial role of AMPARs in synaptic events, including plasticity, neuronal maturation, memory formation, and synaptogenesis.^45^, ^46^ Given this understanding, enhancing AMPAR function using AMPA potentiators has emerged as a potential therapeutic approach for schizophrenia. In line with this hypothesis, our study proves that LT‐102 treatment effectively enhances LTP at hippocampal CA1 synapses. Furthermore, our findings suggest that the effect of LT‐102 on enhancing synaptic strength is due to increased postsynaptic AMPAR‐mediated responses and presynaptic probability of transmitter release. These results further support the potential of LT‐102 as a treatment for CIAS due to modulating synaptic plasticity and influencing cognitive processes.

Schizophrenia patients often exhibit prolonged NMDAR hypofunction, leading to impaired synaptic plasticity and cognitive functioning.^47^ To evaluate the potential therapeutic effects of LT‐102 in vivo, we utilized a schizophrenia model induced by PCP, an NMDAR antagonist known to replicate cognitive impairments observed in schizophrenia patients.^48^ Consistent with previous research,^49^ chronic administration of PCP resulted in significant spatial and recognition memory impairments in mice, as evidenced by their performance in the Morris water maze and novel object recognition tests. In contrast, treatment with LT‐102 in a dose‐dependent manner effectively ameliorated these deficits in both cognitive tests. LT‐102 ameliorates memory deficits in the PCP‐induced schizophrenia model, highlighting its potential therapeutic effects for CIAS.

BDNF is a widely distributed neurotrophin that plays a crucial role in synaptic plasticity and cognitive function.^50^ Research consistently indicates that peripheral BDNF levels are significantly lower in individuals with schizophrenia.^51^ As a result, BDNF has been considered a biological marker of cognitive functioning in schizophrenia^52^ and is a valuable tool for evaluating cognitive enhancement approaches in this disorder.^53^ AMPARs consist of four subunits, including GluA1‐4. Among them, GluA1, a subtype of AMPARs that allows calcium permeability, plays a vital role in various forms of synaptic plasticity.^54^ Prior evidence has shown that decreased hippocampal GluA1 mRNA and protein in the hippocampal tissue of schizophrenia patients^55^, ^56^, ^57^ may be causally linked to impairments in selective attention and memory.^58^, ^59^ In our study, LT‐102 treatment led to an agonist‐dependent increase in BDNF expression and GluA1 phosphorylation in primary neurons. Moreover, PCP‐treated mice exhibited downregulation of BDNF along with decreased GluA1‐Ser831 phosphorylation, while LT‐102 treatment reversed these deficits in a dose‐dependent manner. We postulate that an overall improvement in learning and memory observed in our study may be related to the restoration of BDNF expression and GluA1 phosphorylation at Ser831. These findings highlight the potential mechanisms underlying the cognitive benefits of LT‐102 treatment in schizophrenia. By promoting BDNF expression and enhancing GluA1 phosphorylation, LT‐102 may contribute to the restoration of synaptic plasticity and cognitive function in individuals with schizophrenia.

## 5 LIMITATIONS

We acknowledge several limitations of our study. Firstly, comprehensive toxicological and safety assessments of LT‐102 should be conducted to ensure its safety profile. Secondly, this study lacks a positive control to better assess the benefits of LT‐102. Lastly, the current animal model, which manipulates the N‐methyl‐D‐aspartic acid receptor, focuses solely on the cognitive deficits of schizophrenia and does not fully replicate the complex and heterogeneous etiology of this disease. Hence, it is essential to explore alternative animal models of schizophrenia that can accurately replicate both the ‘positive‐like’ symptoms using the prepulse inhibition test and the ‘negative‐like’ symptoms through the social interaction test. These additional models will allow for a more thorough assessment of the therapeutic impacts of LT‐102. In addition, future studies are needed to assess the effects of LT‐102 on CaMK2b activity, given the significance of CaMK2b activity in cognitive tasks.

## 6 CONCLUSION

In summary, our study highlights the potential of LT‐102 as a potent and selective AMPAR potentiator with minimal agonistic effect. LT‐102 treatment enhances synaptic plasticity and effectively reverses cognitive impairments induced by subchronic PCP exposure, making it a promising candidate for CIAS treatment. Moreover, gaining a comprehensive understanding of the mechanisms underlying the therapeutic effects of LT‐102 will facilitate its progression into clinical trials.

## AUTHOR CONTRIBUTIONS

Tao Li and Xueyu Qi conceived and designed the study. Xueyu Qi, Xueli Yu, Long Wei, Han Jiang, Jiangwen Dong, and Hongxing Li performed the experiments. Xueyu Qi, Xueli Yu, Long Wei, Yingying Wei, and Liansheng Zhao analyzed and interpreted the data. Xueyu Qi, Xueli Yu, Wei Deng, Wanjun Guo, Xun Hu, and Tao Li wrote the manuscript. All authors revised the manuscript critically and approved the final version.

## FUNDING INFORMATION

This work was partly funded by National Natural Science Foundation of China (81920108018, 82371524, and 82371503), Key R&D Program of Zhejiang Province (2022C03096), Special Foundation for Brain Research from Science and Technology Program of Guangdong Province (2018B030334001), Natural Science Foundation of Zhejiang Province (LY22H090009), Clinical Research Innovation Project, West China Hospital, Sichuan University (2019HXCX02), and Project for Hangzhou Medical Disciplines of Excellence & Key Project for Hangzhou Medical Disciplines.

## CONFLICT OF INTEREST STATEMENT

The authors declare no competing interests.

## Supporting information


Figure S1.

Data S1.

## Data Availability

All processed data used in this study can be obtained from the corresponding author upon reasonable request.
