# Supplementary figures and images for: Novel α‐amino‐3‐hydroxy‐5‐methyl‐4‐isoxazole‐propionic acid receptor (AMPAR) potentiator LT‐102: A promising therapeutic agent for treating cognitive impairment associated with schizophrenia
Source: CNS Neurosci Ther. 2024 Apr 14;30(4):e14713. doi: 10.1111/cns.14713 (PMC11016348; doi:10.1111/cns.14713)

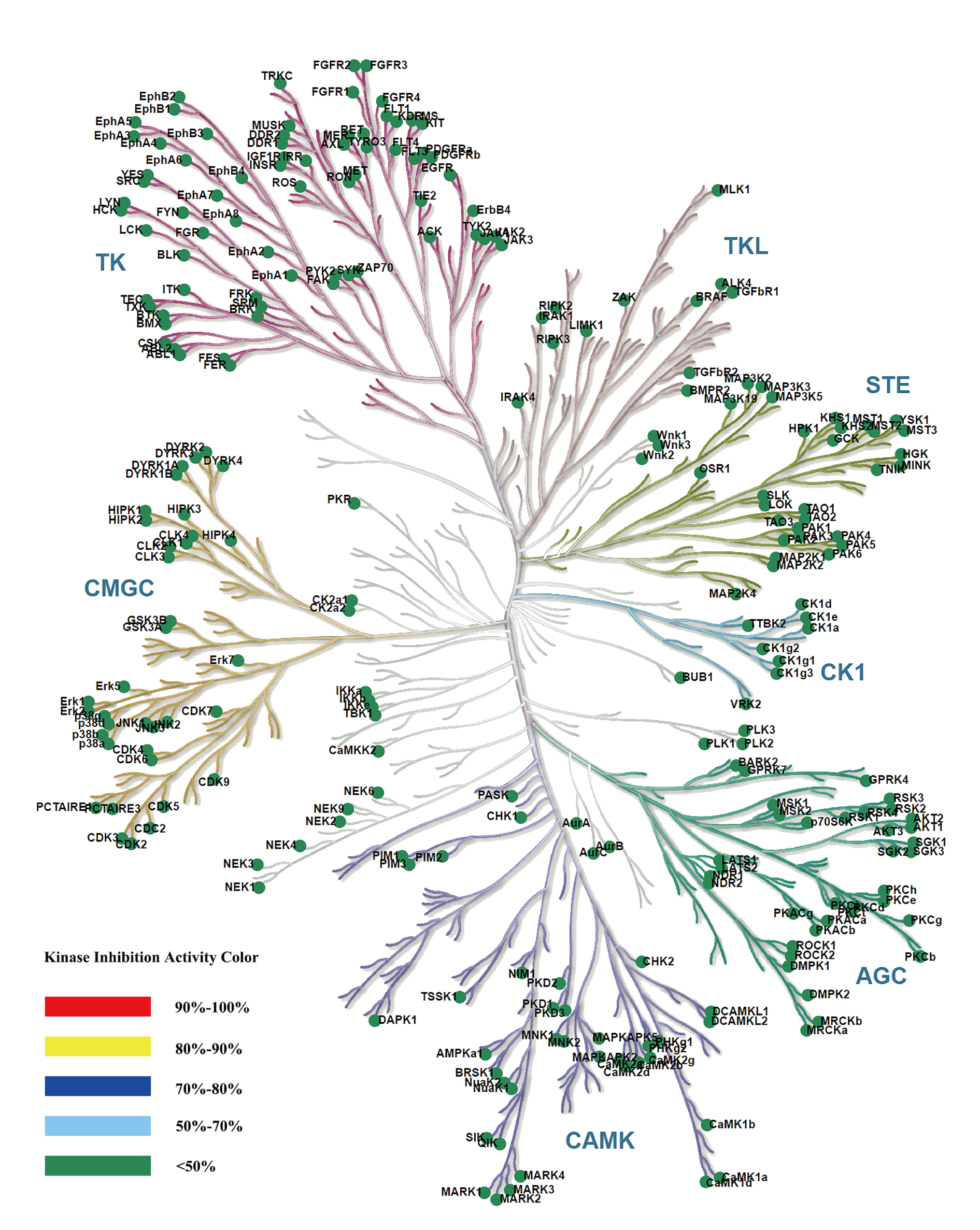

Supplement: Supplementary file 1 — Figure S1. [file CNS-30-e14713-s002.tif]
