# Supplementary material for: Novel α‐amino‐3‐hydroxy‐5‐methyl‐4‐isoxazole‐propionic acid receptor (AMPAR) potentiator LT‐102: A promising therapeutic agent for treating cognitive impairment associated with schizophrenia
Source: CNS Neurosci Ther. 2024 Apr 14;30(4):e14713. doi: 10.1111/cns.14713 (PMC11016348; doi:10.1111/cns.14713)

Supplementary Data for

Novel α-amino-3-hydroxy-5-methyl-4-isoxazole-propionic Acid Receptor (AMPAR) Potentiator LT-102: A Promising Therapeutic Agent for Treating Cognitive Impairment Associated with Schizophrenia

Xueyu Qi [1, 2, 3], Xueli Yu [1, 2, 3], Long Wei [1], Han Jiang [1], Jiangwen Dong [1], Hongxing Li [1], Yingying Wei [4], Liansheng Zhao [4], Wei Deng [1, 2, 3], Wanjun Guo [1, 2, 3], Xun Hu [5], and Tao Li[1, 2, 3]

[1] Affiliated Mental Health Center & Hangzhou Seventh People's Hospital and School of Brain Science and Brain Medicine, Zhejiang University School of Medicine, Hangzhou, 310058, China

[2] Liangzhu Laboratory, MOE Frontier Science Center for Brain Science and Brain-machine Integration, State Key Laboratory of Brain-machine Intelligence, Zhejiang University, 1369 West Wenyi Road, Hangzhou 311121, China

[3] NHC and CAMS Key Laboratory of Medical Neurobiology, Zhejiang University, Hangzhou 310058, China

[4] The Psychiatric Laboratory, the State Key Laboratory of Biotherapy, West China Hospital of Sichuan University, Chengdu, Sichuan, China

[5] The Clinical Research Center and Department of Pathology, The Second Affiliated Hospital, Zhejiang University School of Medicine, Hangzhou, Zhejiang, China

Correspondence: Tao Li, Affiliated Mental Health Center & Hangzhou Seventh People's Hospital, Zhejiang University School of Medicine, Hangzhou, Zhejiang 310013, P.R. China.
Tel: 0086-571-85121532
Email: litaozjusc@zju.edu.cn

Funding information: National Natural Science Foundation of China, Grant/Award Number: 81920108018, 82371524 and 82371503; Key R&D Program of Zhejiang Province, Grant/Award Number: 2022C03096; Special Foundation for Brain Research from Science and Technology Program of Guangdong Province, Grant/Award Number: 2018B030334001; Natural Science Foundation of Zhejiang Province, Grant/Award Number: LY22H090009; Clinical Research Innovation Project, West China Hospital, Sichuan University, Grant/Award Number: 2019HXCX02; Project for Hangzhou Medical Disciplines of Excellence & Key Project for Hangzhou Medical Disciplines.

These authors contributed equally: Xueyu Qi, Xueli Yu, and Long Wei

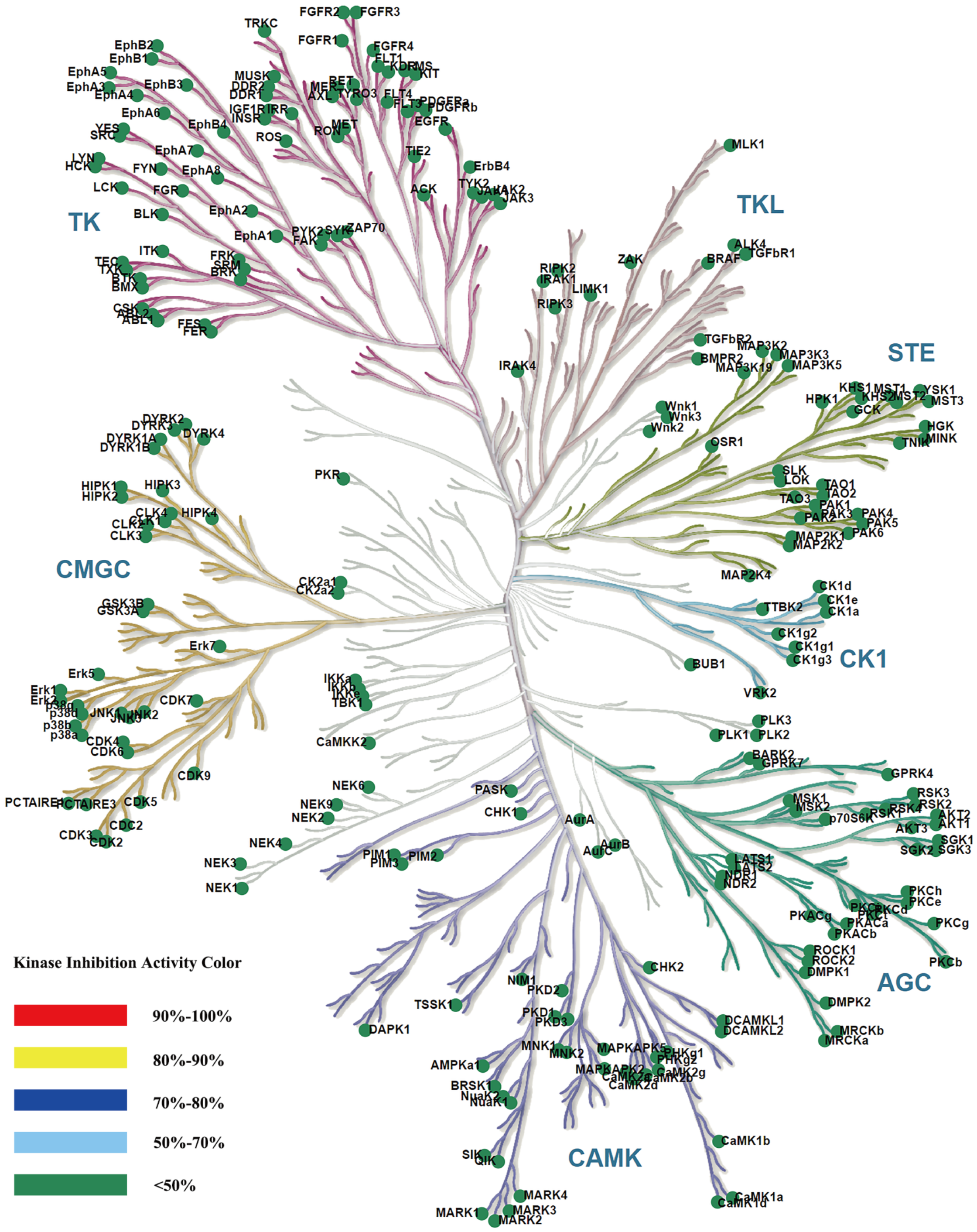

FIGURE S1: TREEspot dendrogram depicting the selectivity profile of LT-102 (10 μM) tested against 310 human kinases. A concentration of 10 μM was used for the measurements, and affinity was defined as a percentage of the DMSO control (% control).

Table S1: X-Ray data collection and refinement statistics.

hGluA2/LT-102<br>(PDB code: **8I0B**)	
**Data collection**	
Space Group	P21221
**Cell dimensions**	
Unit Cell (Å)	121.40 47.10 98.41
Unit Cell (°)	90.0, 90, 90
Wavelength (Å)	0.9785
Resolution (Å)	98.41-1.73
R_{merge}	0.048 (0.858)
$CC_{1/2}$	99.8 (89.5)
I/sigma	37.6 (2.7)
Completeness (%)	99.4 (96.6)
Redundancy	11.1 (10.8)
**Structure refinement**	
Number of measured reflections	712336
Number of unique reflections	59162
R_{work} / R_{free} (%)	22.06/27.81
No. atoms	4525
Protein	4072
Water	381
Ligand	72
Average B value ($Å^2$)	91.33
Protein	87.34
Water	55.99
Others	91.33
**R.m.s. deviations**	
Bonds (Å)	0.012
Angle (°)	1.239
**Ramachandran plot statistics (%)**	
Most favorable	97.68
allowed	2.13
Disallowed	0.19

Table S2: Inhibition activities of LT-102 against 310 kinases.

**Kinase**	**LT-102 (10μM) (%inhibition)**<br>**Data1**	**LT-102 (10μM) (%inhibition)**<br>**Data2**	**STDEV**	**LT-102**<br>**(Ave_%inhibition @10 μM)**
RET S891A	49.15	48.5	0.46	48.82
PHKG1	56.25	39.38	11.93	47.81
TNIK	51.64	42.87	6.2	47.26
RET V804L	45.28	49.01	2.64	47.15
PIK3CA	49.25	44.85	3.12	47.05

Kinase	LT-102 (10μM) (%inhibition) Data1	LT-102 (10μM) (%inhibition) Data2	STDEV	LT-102 (Ave_%inhibition @10 μM)
CaMK2β	41.07	52.48	8.07	46.77
PHKG2	43.94	47.06	2.2	45.5
HGK	47.93	37.67	7.25	42.8
CaMK1β	39.96	27.18	9.04	33.57
BTK C481S	34.18	32.65	1.08	33.41
RET G810S	36.48	22.83	9.65	29.65
p38α	30.6	27.17	2.42	28.89
CaMK1δ	30.81	24.7	4.33	27.76
CaMK2δ	24.31	31.04	4.76	27.68
PIK3CB	31.63	19.37	8.67	25.5
TRKC	31.13	16.45	10.38	23.79
MINK	17.17	25.36	5.79	21.26
CAMKK2	15.96	23.01	4.99	19.48
PIM1	29.6	7.82	15.4	18.71
TEC	30.56	6.46	17.04	18.51
PRKCQ	4.87	29.51	17.43	17.19
LATS2	27.9	6.33	15.25	17.11
CaMK2γ	20.97	12.3	6.13	16.63
MLK1	26.8	3.13	16.73	14.97
KIT T670I	18.78	10.62	5.77	14.7
RET V804M	18.01	10.54	5.28	14.28
NuaK1	12.89	15.17	1.61	14.03
BRAF	25.54	1.25	17.17	13.4
RET G810R	29.56	-4.32	23.96	12.62
RSK3	18.28	6.18	8.56	12.23
ABL2	9.95	12.99	2.15	11.47
RET G810C	8.45	14.48	4.26	11.47
DMPK2	10.7	11.74	0.74	11.22
PKACγ	12.13	9.69	1.72	10.91
EGFR V769-D770 insGE	28.21	-6.88	24.82	10.67
MAP2K2	16.33	4.24	8.55	10.29
EPHA3	9.87	9.87	0	9.87
TRKA G595R	11.04	8.27	1.96	9.65
LOK	13.65	5.52	5.75	9.59
LYNb	-3.44	22.57	18.4	9.57
TIE2	3.84	14.95	7.85	9.4
KIT D816V	7.83	9.1	0.89	8.47
ZAK	25.77	-8.86	24.48	8.45
NEK4	9.8	6.7	2.2	8.25
CaMk2α	6.77	9.67	2.05	8.22
CDK18/CycY	10.62	5.7	3.48	8.16
TAOK3	6.56	9.54	2.1	8.05
CLK1	10	5.7	3.04	7.85

Kinase	LT-102 (10μM) (%inhibition) Data1	LT-102 (10μM) (%inhibition) Data2	STDEV	LT-102 (Ave_%inhibition @10 μM)
FYN [b]	4.02	11.49	5.28	7.76
FLT3 D835Y	10.4	5.08	3.76	7.74
PAK4	5.81	9.65	2.72	7.73
KIT	9.77	5.4	3.09	7.59
RIPK2	20.48	-5.57	18.42	7.46
CK1δ	7.96	6.62	0.95	7.29
MRCKβ	5.57	9	2.43	7.29
TYK2	1.53	12.92	8.06	7.22
BRSK1	9.87	3.74	4.33	6.81
BLK	4.35	9.1	3.36	6.73
EGFR D770_N771insNPG	8	4.86	2.22	6.43
MSK2	13	-0.72	9.7	6.14
IKKα	-1.95	13.99	11.27	6.02
KIT V559D	-3.12	14.8	12.67	5.84
DAPK1	5.08	6.54	1.03	5.81
FAK	4.28	7.3	2.14	5.79
DYRK3	3.76	7.34	2.53	5.55
EPHB4	3.32	7.54	2.99	5.43
PASK	4.57	5.38	0.58	4.97
DDR2	4.75	5.13	0.27	4.94
AurC	4.71	4.99	0.2	4.85
JAK1	4.63	4.57	0.05	4.6
PKACβ	5.8	3.39	1.7	4.6
TXK	7.85	0.8	4.99	4.32
Erk5	5.8	2.85	2.08	4.32
JNK2	2	6.56	3.23	4.28
SRM	8.3	0.13	5.78	4.21
CDK4/CycD3	1.07	7.22	4.35	4.15
FGFR2 V564F	6.02	2.11	2.76	4.07
SRC	5.91	1.98	2.78	3.94
ROCK1	6.6	1.29	3.76	3.94
PRKCG	13.1	-5.35	13.05	3.87
CK2α2/β	3.83	3.71	0.08	3.77
FGFR3	4.08	3.39	0.49	3.73
IGF1R	5.35	2.12	2.29	3.73
EPHA5	3.24	3.71	0.34	3.48
EGFR d746-750 T790M C797S	6.72	-0.3	4.97	3.21
FGFR1	4.5	1.82	1.9	3.16
ABL1	3.82	2.4	1	3.11
RSK2	4.22	1.96	1.6	3.09
EPHA4	1.98	3.95	1.39	2.96
CDK3/CycE1	2.8	3.08	0.19	2.94

Kinase	LT-102 (10μM) (%inhibition) Data1	LT-102 (10μM) (%inhibition) Data2	STDEV	LT-102 (Ave_%inhibition @10 μM)
CK1γ2	5.17	0.68	3.17	2.92
JNK3	9.01	-3.36	8.74	2.83
NEK1	4.14	1.51	1.86	2.83
ROCK2	1.01	4.62	2.55	2.82
EPHB2	0.61	4.99	3.09	2.8
FGFR3 V555M	4.14	1.45	1.9	2.79
PGK	4.89	0.57	3.05	2.73
GSK3B	-0.74	6.05	4.81	2.65
AKT3	4.58	0.66	2.77	2.62
CK1γ1	8.1	-2.93	7.8	2.59
JNK1	-8.64	13.78	15.85	2.57
AurA	3.29	1.83	1.04	2.56
KDR	3.32	1.64	1.19	2.48
RET	4.44	0.36	2.89	2.4
JAK3	1.71	2.91	0.85	2.31
p38δ	-1.48	6.07	5.34	2.3
FGFR2	2.64	1.85	0.56	2.24
DYRK2	3.61	0.83	1.97	2.22
FGFR1 V561M	1.12	3.16	1.44	2.14
HCK	-1.39	5.52	4.89	2.06
ROS1	2.99	1.03	1.39	2.01
CDK6/CycD3	-0.4	3.94	3.07	1.77
EGFR d746-750	1.05	2.49	1.02	1.77
TSSK1	6.66	-3.28	7.03	1.69
FLT3	0.14	3.21	2.17	1.68
PKACα	0.99	2.34	0.95	1.66
LCK	0.26	3.06	1.99	1.66
P70s6k	2.87	0.41	1.74	1.64
EGFR T790M C797S L858R	2.4	0.82	1.12	1.61
PAK2	2.58	0.63	1.38	1.61
CDK5/p35NCK	5.41	-2.21	5.39	1.6
DYRK1B	-0.19	3.39	2.53	1.6
EPHA7	0.75	2.42	1.18	1.59
p38γ	0.48	2.61	1.5	1.55
YES	5.01	-2.04	4.98	1.48
CK2α1/β	0.71	2.06	0.95	1.38
IRR	2.14	0.6	1.09	1.37
FGR	1.79	0.9	0.63	1.34
MET Y1230C	1.48	1.13	0.25	1.31
EGFR	-9.05	11.51	14.54	1.23
EGFR L858R	1.26	1.04	0.16	1.15
RET Y806H	2.47	-0.31	1.96	1.08
LYNa	2.59	-0.58	2.24	1.01

Kinase	LT-102 (10μM) (%inhibition) Data1	LT-102 (10μM) (%inhibition) Data2	STDEV	LT-102 (Ave_%inhibition @10 μM)
FRK	0.42	1.56	0.8	0.99
MAP3K3	2.08	-0.17	1.59	0.95
PKD2	2.82	-0.95	2.67	0.94
FGFR2 K641R	7.12	-5.37	8.83	0.87
PYK2	1.13	0.53	0.42	0.83
EPHB3	2.55	-1.05	2.55	0.75
RET M918T	5.55	-4.07	6.81	0.74
CDK2/CycA2	0.62	0.83	0.15	0.73
SYK	1.34	-0.08	1	0.63
RSK1	2.56	-1.42	2.82	0.57
EPHA1	1.47	-0.43	1.34	0.52
TAOK2	-3.91	4.71	6.09	0.4
CHK1	-0.73	1.46	1.55	0.36
MAPKAPK2	-0.78	1.44	1.57	0.33
PDGFRβ	-0.55	1.08	1.15	0.27
AKT2	-0.8	1.29	1.48	0.25
BRSK1	7.12	-6.63	9.72	0.25
SGK3	0.05	0.43	0.27	0.24
EGFR T790M C797S	2.1	-1.68	2.67	0.21
FES	-0.28	0.56	0.59	0.14
EGFR T790M L858R	-0.2	0.16	0.25	-0.02
FGFR2 N549H	-2.14	2.07	2.97	-0.04
Erk2	-2.74	2.64	3.81	-0.05
PKD1	0.57	-0.91	1.05	-0.17
RON	-1.54	1.11	1.87	-0.22
EPHB1	2.05	-2.53	3.24	-0.24
AKT1	-0.92	0.43	0.96	-0.24
CSF1R	-0.27	-0.24	0.02	-0.26
FLT4	-1	0.44	1.02	-0.28
DYRK4	-0.6	0.03	0.45	-0.29
PDGFRα	-1.63	1.05	1.9	-0.29
PAK5	-1.05	0.26	0.92	-0.39
TYRO3	-0.56	-0.23	0.23	-0.39
FGFR3 K650E	0.25	-1.25	1.06	-0.5
CHK2	-6.23	4.92	7.88	-0.66
PAK1	-1.52	0.19	1.21	-0.67
EML4-ALK	22.92	-24.4	33.46	-0.74
TAOK1	-17.56	16.07	23.78	-0.75
RIPK3	17.71	-19.3	26.17	-0.8
TTBK2	7.63	-9.33	11.99	-0.85
PAK3	-0.8	-1.01	0.15	-0.91
INSR	-1.83	-0.07	1.24	-0.95

Kinase	LT-102 (10μM) (%inhibition) Data1	LT-102 (10μM) (%inhibition) Data2	STDEV	LT-102 (Ave_%inhibition @10 μM)
MST3	0.69	-3.14	2.71	-1.23
FYN [a]	1.44	-4.18	3.97	-1.37
IKKε	1.11	-3.92	3.56	-1.4
SGK	-2.68	-0.14	1.79	-1.41
MUSK	-0.98	-1.88	0.64	-1.43
MAP3K19	2.83	-5.96	6.22	-1.56
EGFR T790M	0.33	-3.66	2.82	-1.66
SGK2	-1.95	-1.38	0.4	-1.67
DDR1	-2.44	-0.97	1.04	-1.7
ACK	-1.38	-2.07	0.49	-1.73
Her4	-2.39	-1.33	0.75	-1.86
EGFR C797S	-1.37	-2.34	0.69	-1.86
DCAMKL2	-1.6	-2.32	0.51	-1.96
BRK	10.6	-14.65	17.85	-2.03
JAK2	-1.13	-3.12	1.41	-2.13
CLK2	20.31	-24.77	31.88	-2.23
AXL	-5.69	0.81	4.6	-2.44
MET D1228H	0.17	-5.09	3.72	-2.46
FLT3 ITD	-3.18	-1.88	0.92	-2.53
PAK6	-4.48	-0.6	2.74	-2.54
CDK1/CycA2	1.79	-6.94	6.17	-2.58
CK1γ3	0.32	-5.51	4.13	-2.59
DYRK1A	-6.53	1.31	5.54	-2.61
YSK1	1.53	-6.96	6	-2.72
CDK9/CycT1	-3.18	-2.47	0.5	-2.82
MARK2	-7.64	1.92	6.76	-2.86
MSK1	4.76	-10.82	11.02	-3.03
MAP3K5	-2.8	-3.3	0.36	-3.05
PRKCH	-8.23	1.87	7.14	-3.18
DMPK1	-19.19	12.76	22.59	-3.22
MET Y1230A	-2.7	-3.94	0.88	-3.32
MET D1228N	0.16	-7.01	5.07	-3.43
Erk1	-1.4	-5.51	2.91	-3.45
CLK4	2.2	-9.28	8.12	-3.54
HPK1	0.03	-7.26	5.15	-3.61
MET Y1230D	-2.89	-4.34	1.02	-3.61
ZAP70	-3.75	-3.57	0.13	-3.66
FLT1	-3.28	-4.22	0.67	-3.75
RSK4	-4.06	-3.45	0.43	-3.75
BMPR2	3.09	-11.04	10	-3.97
EGFR L861Q	-4.42	-3.61	0.57	-4.02
p38β	-5.79	-2.53	2.3	-4.16
CDK5/p25NCK	8.38	-16.76	17.78	-4.19
CK1ε	-4.06	-4.42	0.25	-4.24

Kinase	LT-102 (10μM) (%inhibition) Data1	LT-102 (10μM) (%inhibition) Data2	STDEV	LT-102 (Ave_%inhibition @10 μM)
GSK3A	-4.01	-4.89	0.62	-4.45
LIMK1	1.62	-10.55	8.61	-4.46
MARK4	1.89	-10.84	9	-4.47
ABL1 F317I	-6.22	-2.8	2.42	-4.51
EGFR D746-750 T790M	-8.2	-0.85	5.2	-4.52
TGFβR2	-16.29	7.22	16.62	-4.54
ALK 4	-0.19	-9.13	6.32	-4.66
DCAMKL1	0.31	-10.07	7.34	-4.88
CK1α	-5.25	-4.68	0.4	-4.96
MET	-4.23	-5.99	1.24	-5.11
CDK16/CycY	-13.37	3.13	11.66	-5.12
AMPKα1/β1/γ1	4.22	-14.52	13.26	-5.15
NEK6	-8.95	-1.51	5.26	-5.23
MARK3	-3.53	-7.03	2.47	-5.28
PKR	-5.73	-5.8	0.05	-5.77
HIPK3	-1.83	-10.34	6.02	-6.09
CDK2/CycE1	-7.36	-4.88	1.75	-6.12
PLK3	-5.39	-7.03	1.16	-6.21
WNK2	-1.99	-10.85	6.26	-6.42
NDR2	5.44	-18.7	17.06	-6.63
MAP2K1	-7.36	-6.5	0.61	-6.93
IRAK4	-10.63	-3.27	5.21	-6.95
GPRK7	-7.64	-6.62	0.73	-7.13
MARK1	-4.76	-9.64	3.45	-7.2
PLK2	-4.79	-10.46	4.01	-7.63
BRAF V600E	-18.56	3.17	15.36	-7.7
NuaK2	-8.37	-7.07	0.92	-7.72
MST1	-4.8	-10.71	4.18	-7.75
EPHA6	-14.36	-1.4	9.16	-7.88
IRAK1	-7.67	-8.28	0.43	-7.98
GLK	-2.61	-13.59	7.77	-8.1
MAP2K4	-8	-9.16	0.81	-8.58
NEK9	-4.69	-12.48	5.51	-8.59
WNK3	12.92	-30.62	30.79	-8.85
PKD3	-6.71	-11.31	3.25	-9.01
VRK2	9.02	-27.83	26.06	-9.4
MNK1	-5.7	-13.44	5.47	-9.57
Erk7	-11.7	-7.99	2.62	-9.84
CSK	-11.14	-8.55	1.83	-9.84
FGFR4	-15	-4.69	7.29	-9.85
OSR1	-21.04	1.02	15.6	-10.01
MAP3K2	-13.3	-6.8	4.6	-10.05
EPHA2	-10.9	-9.34	1.1	-10.12

Kinase	LT-102 (10μM) (%inhibition) Data1	LT-102 (10μM) (%inhibition) Data2	STDEV	LT-102 (Ave_%inhibition @10 μM)
TGFβR1 T204D	-3.68	-16.7	9.21	-10.19
PIM2	-14.74	-6.15	6.07	-10.44
EGFR A763_Y764insFQEA	-10.03	-11.18	0.82	-10.61
NEK3	-13.19	-9.88	2.34	-11.54
CDK7/CycH/MAT1	-20.09	-3.05	12.05	-11.57
LATS1	-21.03	-2.76	12.92	-11.89
MAPKAPK5	-17.4	-6.99	7.36	-12.2
HIPK2	-14.9	-9.58	3.76	-12.24
SIK	-11.3	-13.3	1.41	-12.3
PRKCD	-14.44	-10.35	2.9	-12.39
CDK1/CycE1	-14.31	-11.48	2	-12.9
PLK1	-14.34	-11.55	1.97	-12.94
CLK3	-21.8	-5.18	11.75	-13.49
SLK	-14.37	-12.78	1.12	-13.58
ABL1 T315I	-26.86	-0.92	18.34	-13.89
MAP4K2	-17.13	-12.73	3.11	-14.93
MST2	-14.84	-16.24	0.99	-15.54
HIPK1	-12.57	-18.63	4.29	-15.6
EPHA8	-1.27	-30.61	20.75	-15.94
MNK2	-16.81	-17.38	0.4	-17.09
ITK	-12.14	-24	8.39	-18.07
GPRK4	-4.62	-31.72	19.16	-18.17
PKCβ1	-19.8	-16.97	2	-18.38
QIK	-16.89	-20.07	2.25	-18.48
TGFβR1	-18.46	-18.64	0.13	-18.55
MER	-19.17	-18.98	0.13	-19.07
NPM1-ALK	-22.65	-15.75	4.88	-19.2
KHS	-1.54	-37.21	25.22	-19.37
HIPK4	-26.77	-12.25	10.26	-19.51
IKKβ	-15.42	-24.96	6.75	-20.19
NEK2	-20.8	-20.1	0.5	-20.45
BTK	-19.49	-23.65	2.94	-21.57
TBK1	-11.55	-31.99	14.45	-21.77
BARK2	-8.41	-35.84	19.4	-22.12
PRKCI	1.01	-48.26	34.84	-23.63
PRKCE	-26.53	-23.6	2.07	-25.07
FER	-23.79	-27.95	2.94	-25.87
BMX	-29.68	-22.14	5.33	-25.91
NDR1	-13.66	-38.64	17.67	-26.15
PKCβ2	-34.17	-19.22	10.57	-26.69
AurB	-27.42	-27.08	0.24	-27.25
NIM1K	-24.96	-31.5	4.63	-28.23
MRCKα	-12.62	-47.7	24.8	-30.16

Kinase	LT-102 (10μM) (%inhibition) Data1	LT-102 (10μM) (%inhibition) Data2	STDEV	LT-102 (Ave_%inhibition @10 μM)
BUB1	-32.45	-34.16	1.21	-33.3
PIM3	-41.05	-31.11	7.03	-36.08
CaMK1α	-43.59	-47.01	2.42	-45.3
WNK1	-54.79	-41.87	9.13	-48.33
ALK G1202R	-64.85	-61.34	2.48	-63.09

Original gel/blot images

The original Western Blots (WB) images are presented below, with the cropped sections highlighted by blue rectangles in the figures.

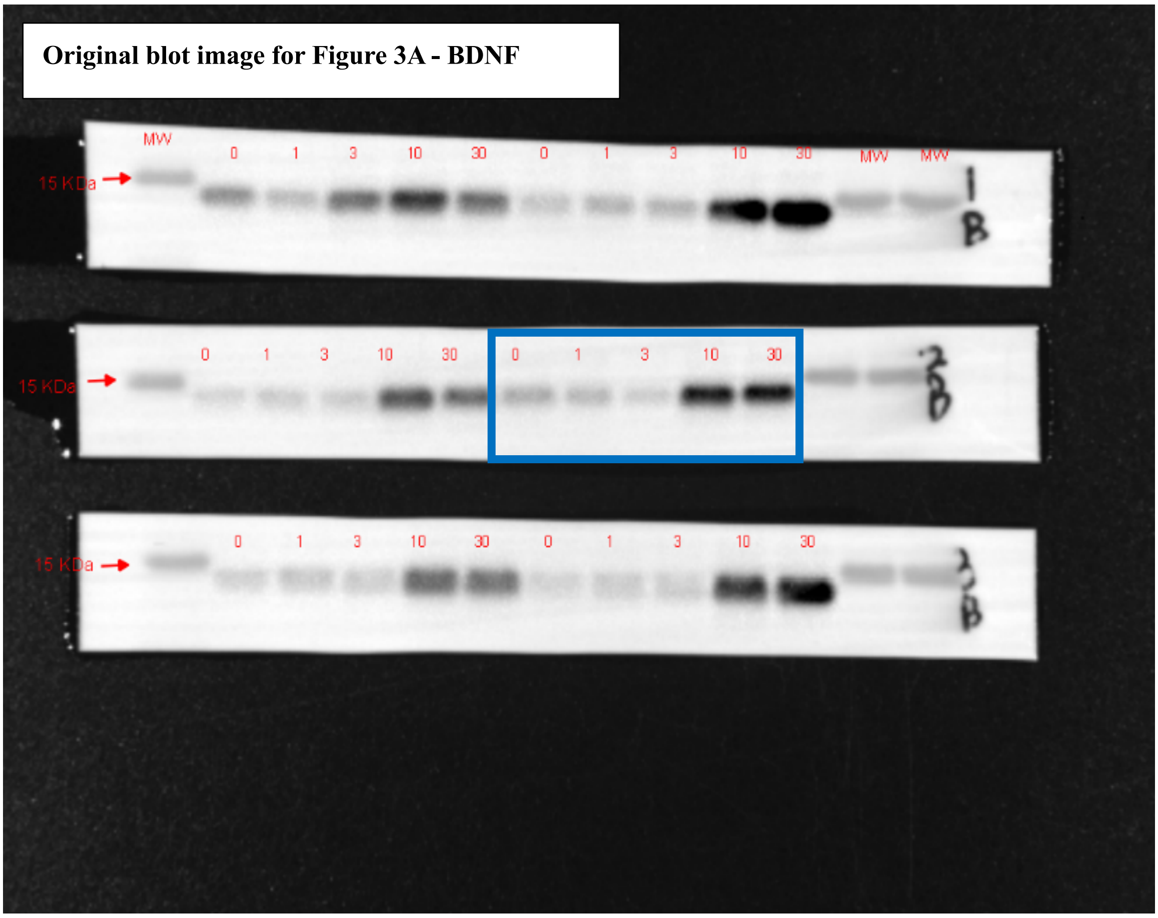

Original blot image for Figure 3A - BDNF

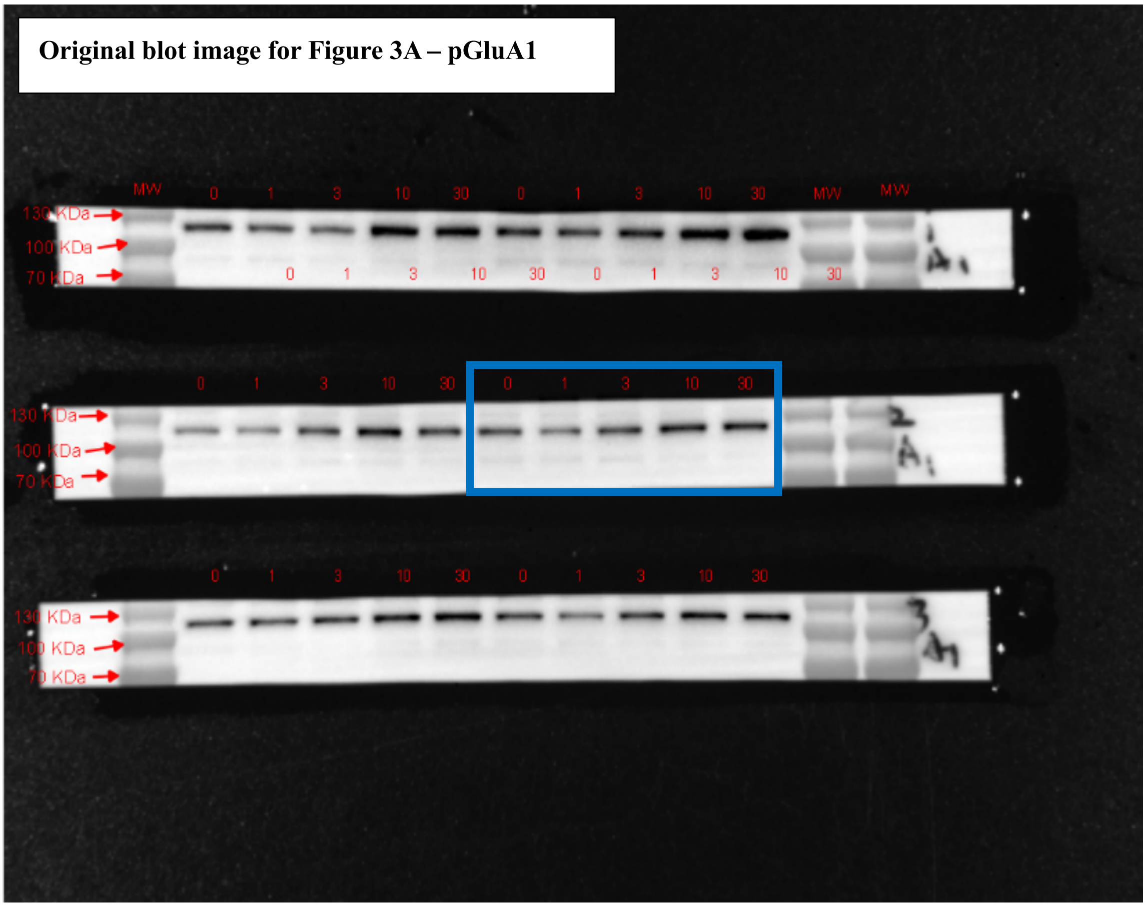
Original blot image for Figure 3A – pGluA1
MW
0 1 3 10 30 0 1 3 10 30
MW MW
130 KDa
100 KDa
70 KDa

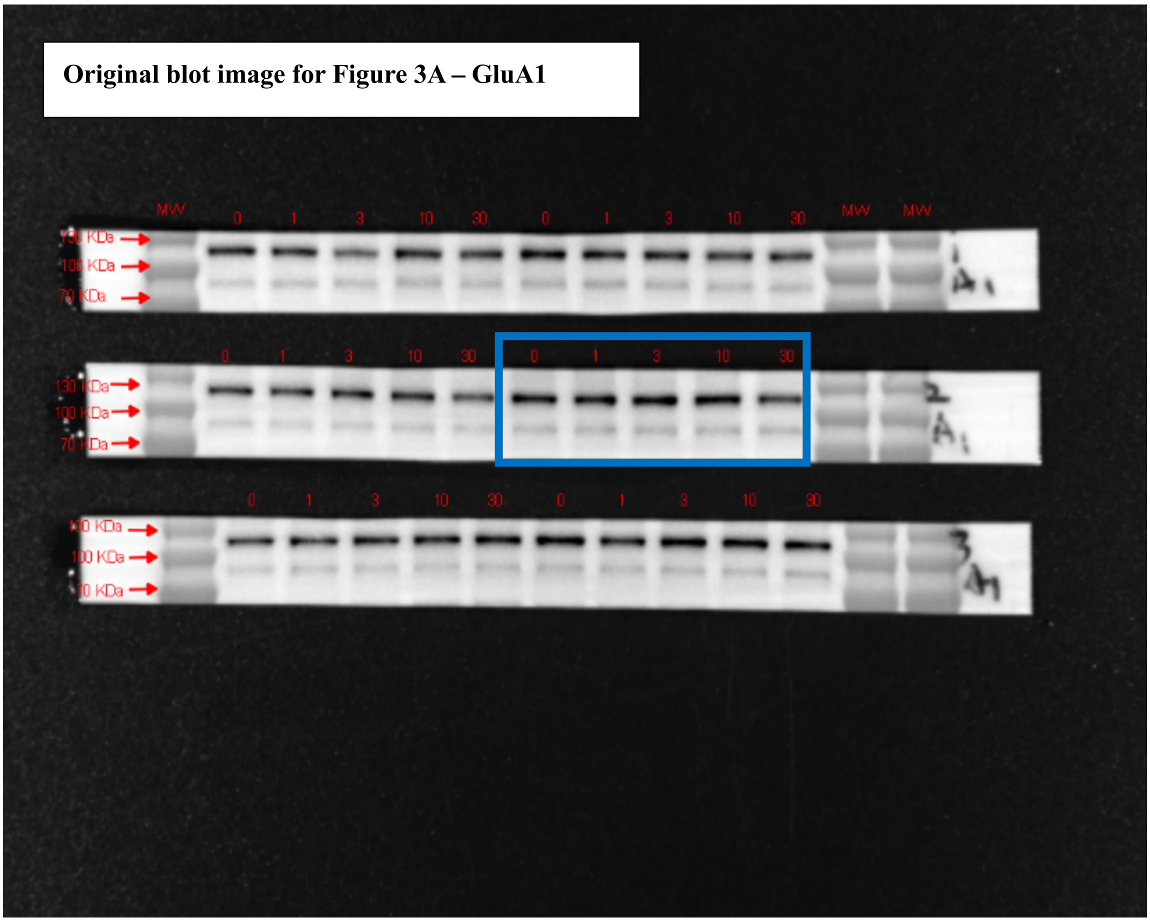
Original blot image for Figure 3A – GluA1
MW
0 1 3 10 30 0 1 3 10 30
MW MW
130 KDa
100 KDa
70 KDa

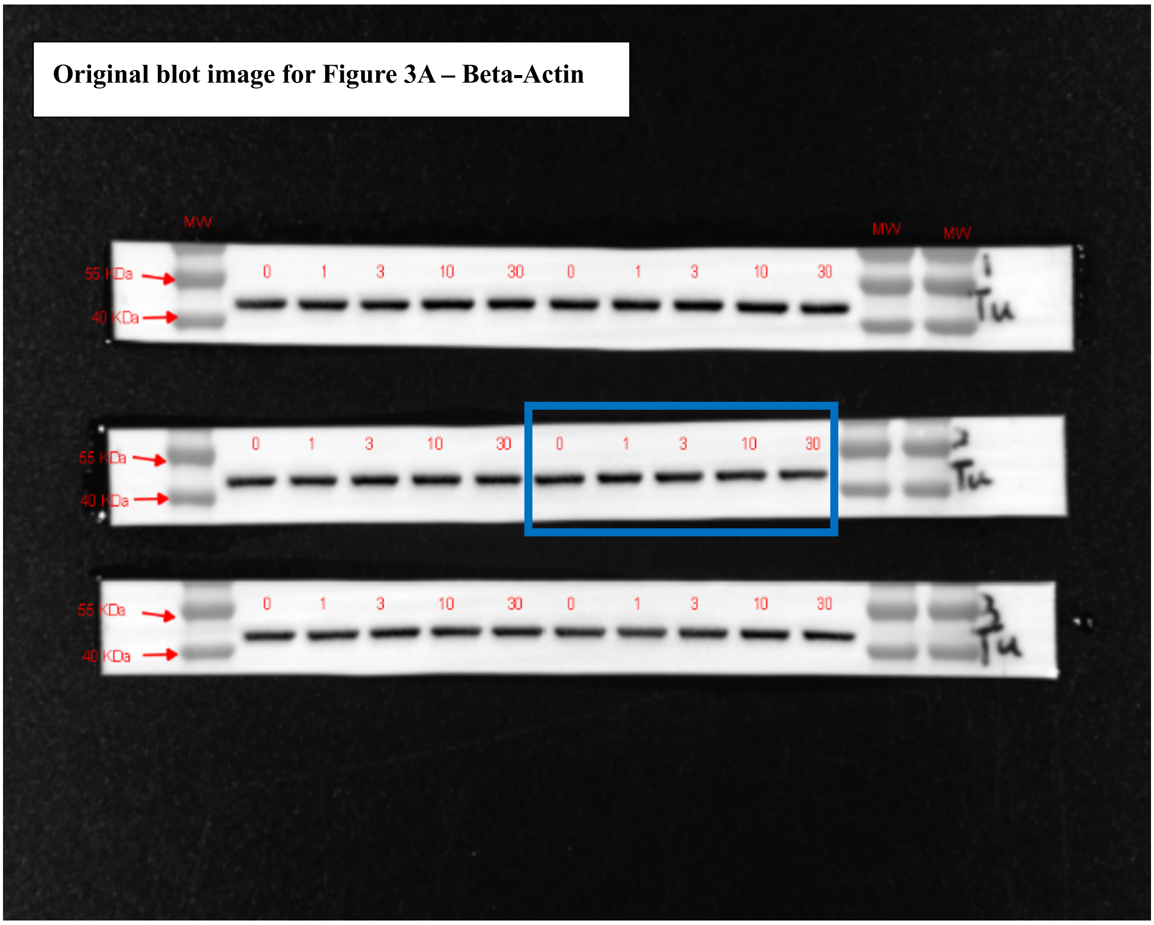

Original blot image for Figure 3A – Beta-Actin

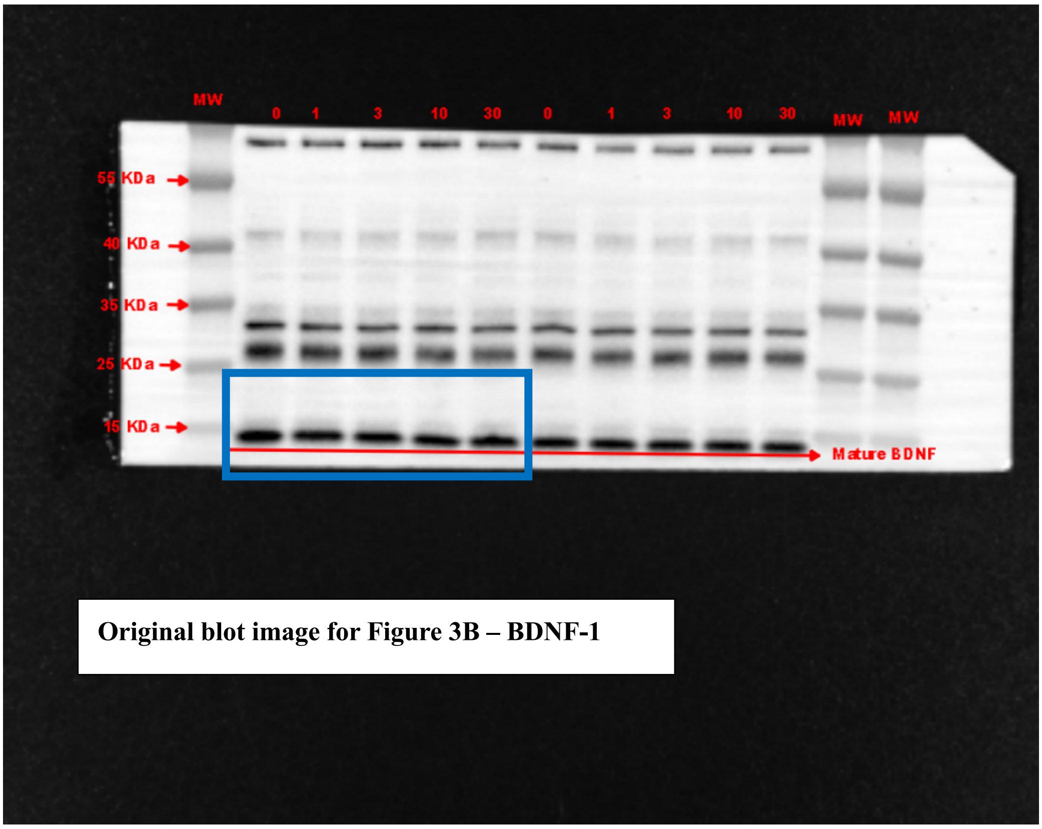

Original blot image for Figure 3B – BDNF-1

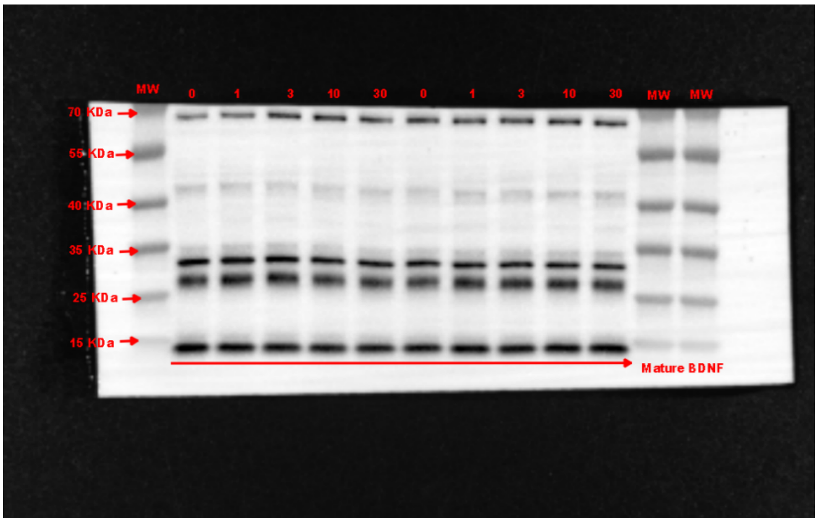

Original blot image for Figure 3B – BDNF-2

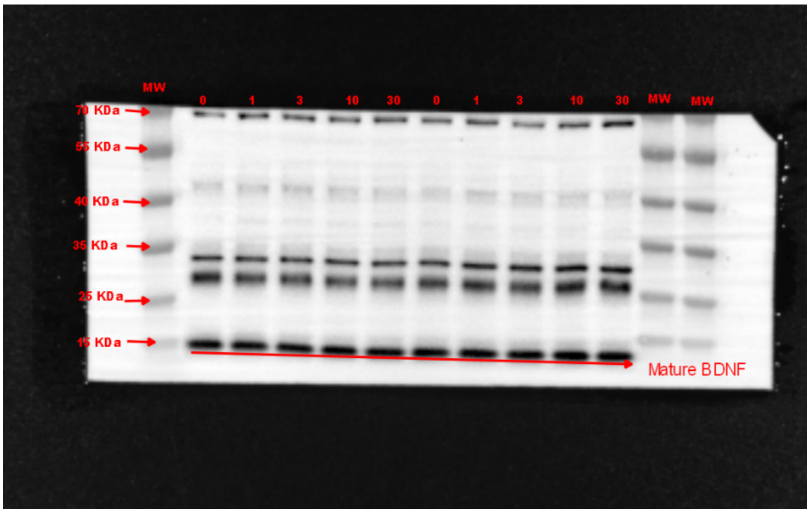

Original blot image for Figure 3B – BDNF-3

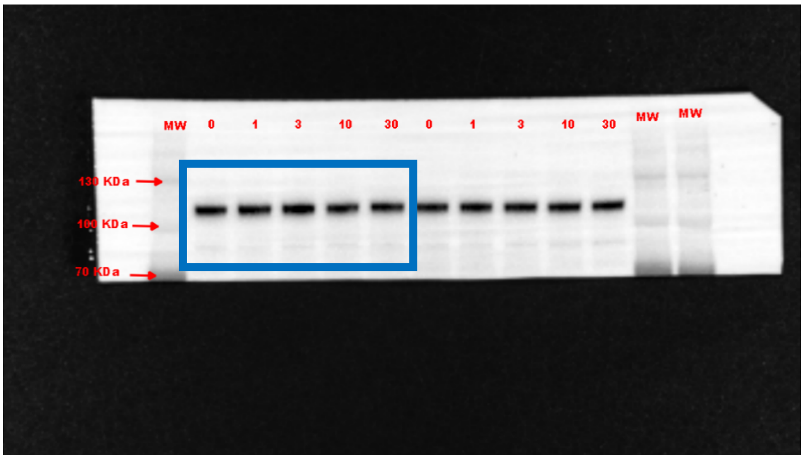

Original blot image for Figure 3B – pGluA1-1

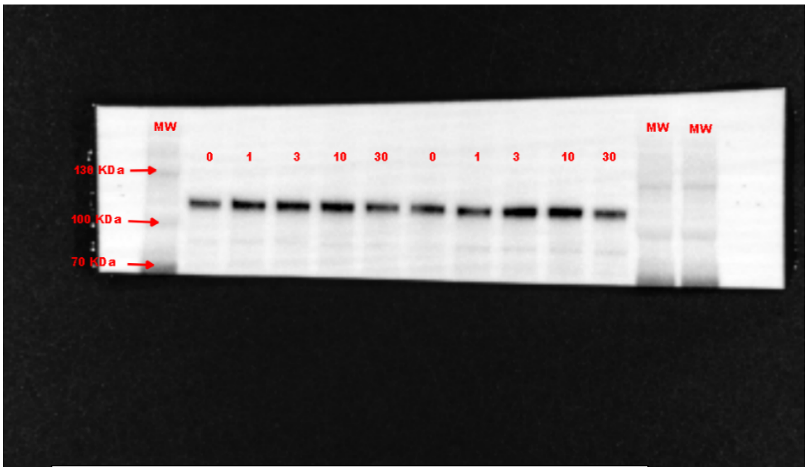

Original blot image for Figure 3B – pGluA1-2

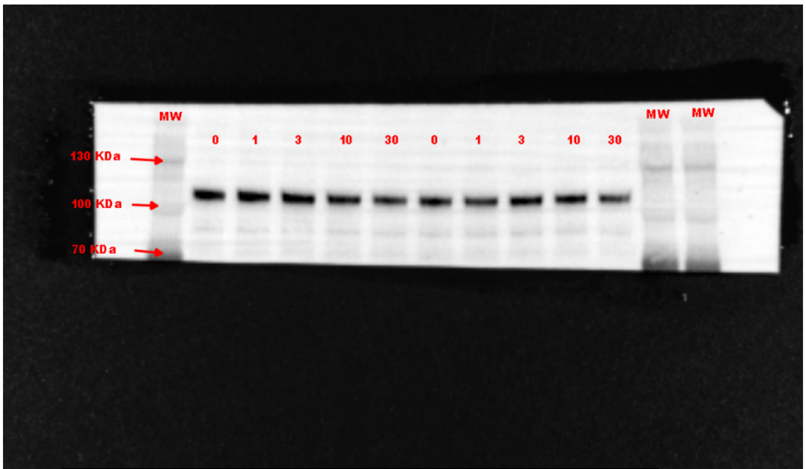

Original blot image for Figure 3B – pGluA1-3

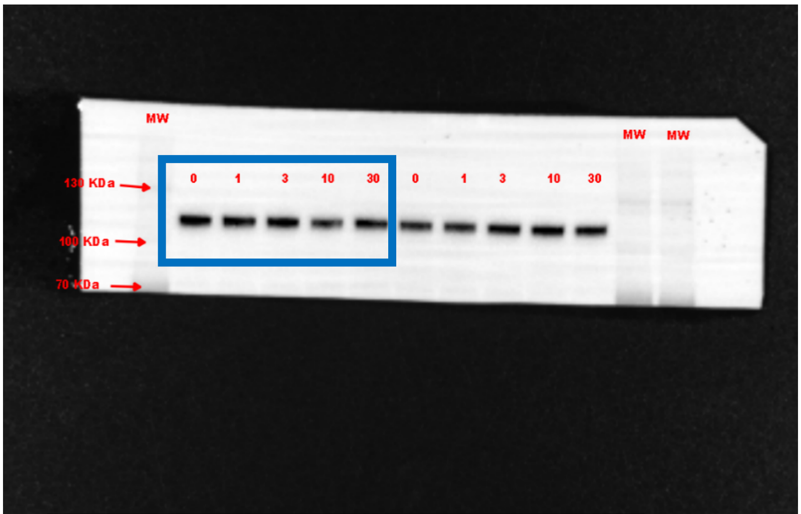

Original blot image for Figure 3B – GluA1-1

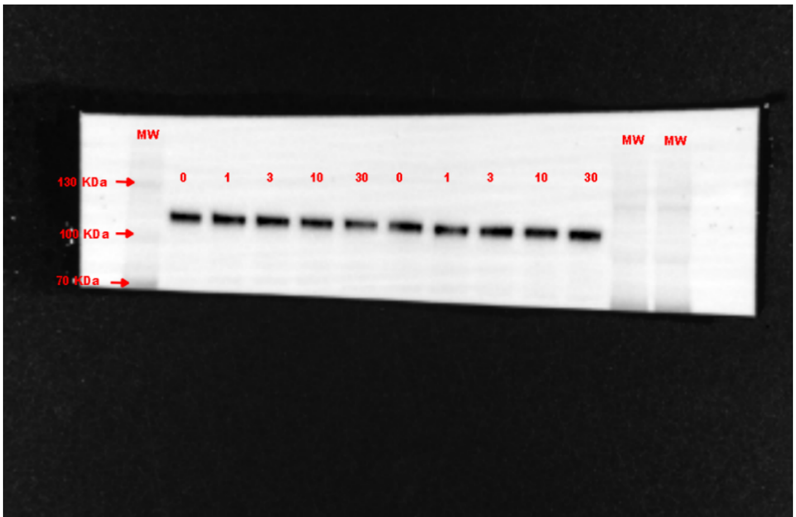

Original blot image for Figure 3B – GluA1-2

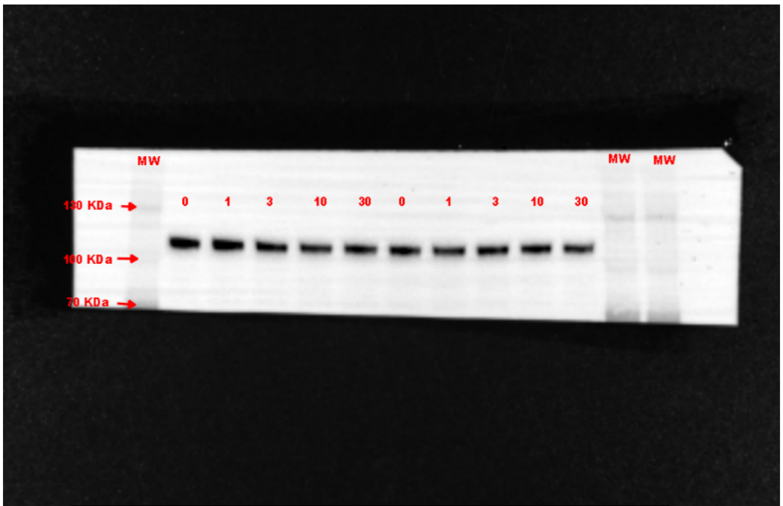

Original blot image for Figure 3B – GluA1-3

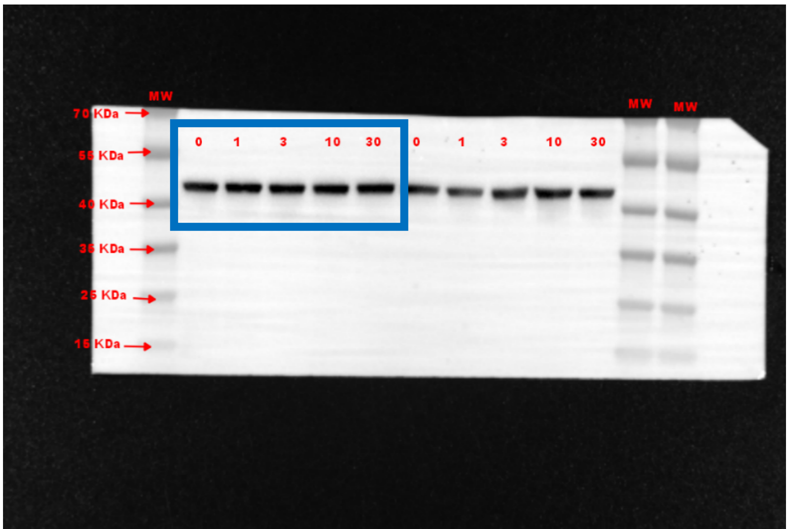

Original blot image for Figure 3B – Beta-Actin-1

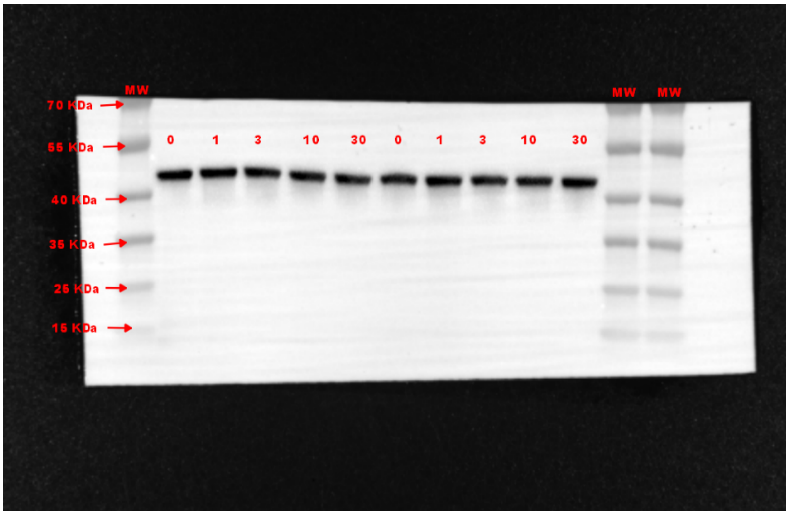

Original blot image for Figure 3B – Beta-Actin-2

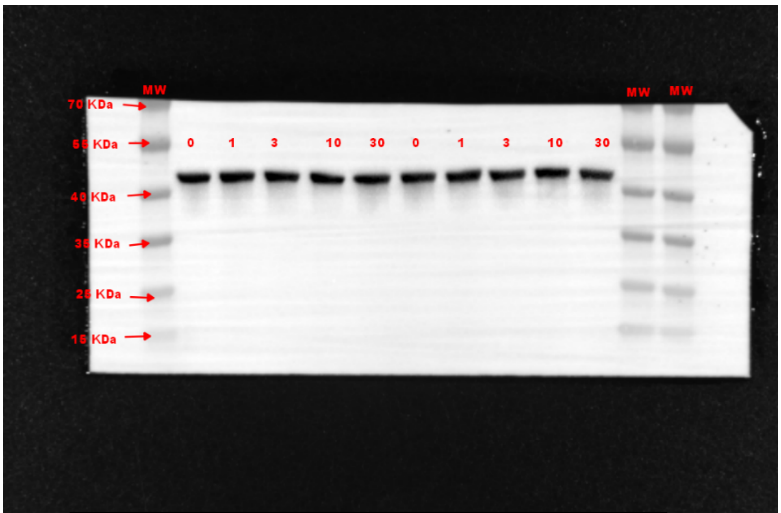

Original blot image for Figure 3B – Beta-Actin-3

Original blot image for Figure 3C – BDNF-1

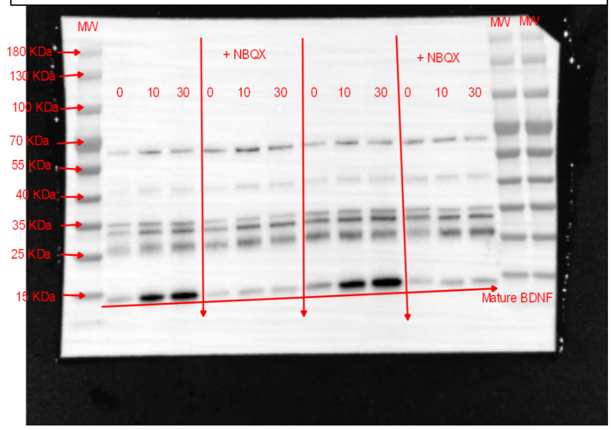

Original blot image for Figure 3C – Beta-Actin for BDNF-1

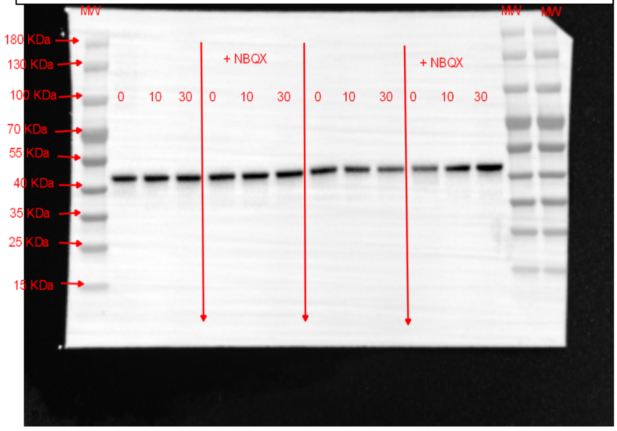

Original blot image for Figure 3C – BDNF-2

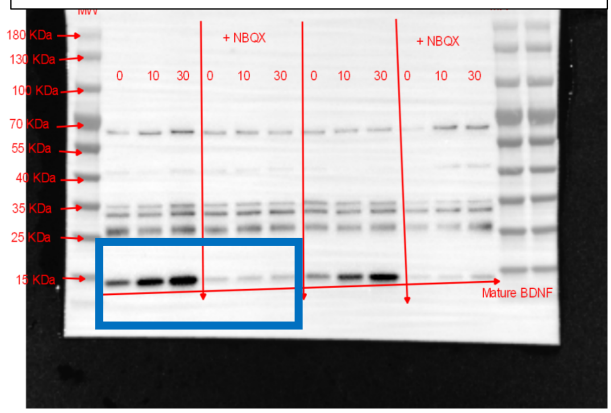

Original blot image for Figure 3C – Beta-Actin for BDNF-2

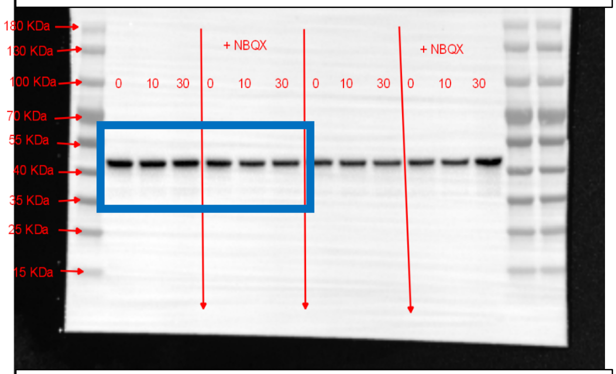

Original blot image for Figure 3C – BDNF-3

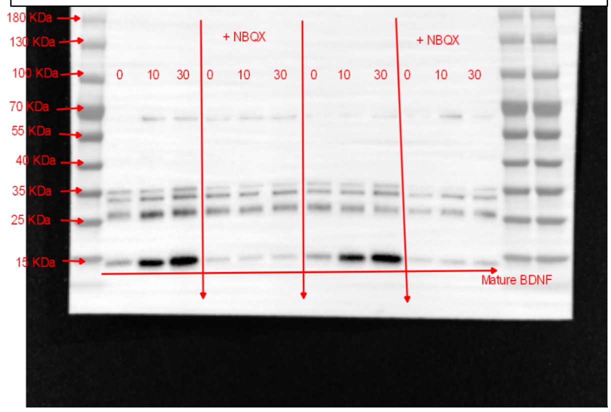

Original blot image for Figure 3C – Beta-Actin for BDNF-3

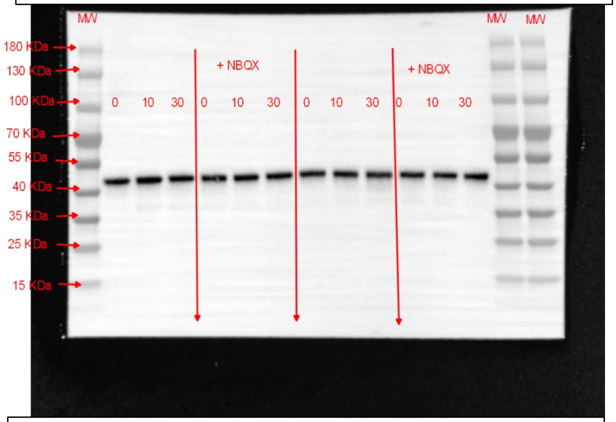

Original blot image for Figure 3C – pGluA1-1

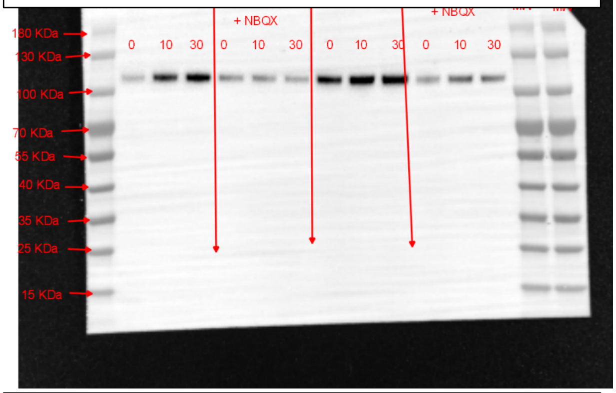

Original blot image for Figure 3C – Beta-Actin for pGluA1-1

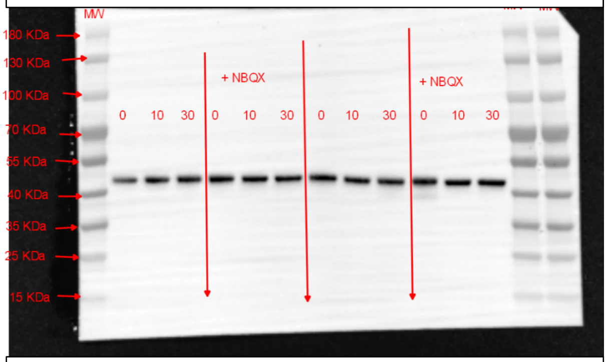

Original blot image for Figure 3C – pGluA1-2

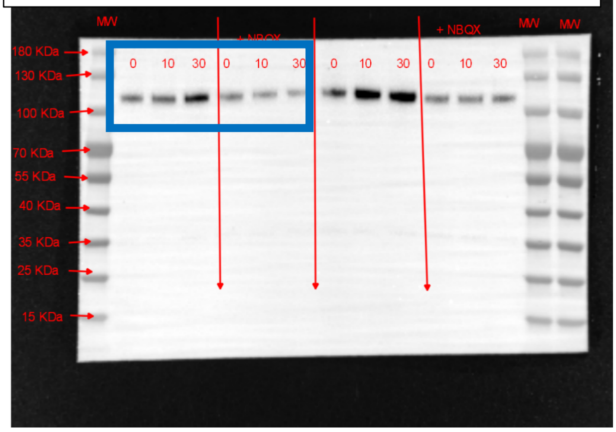

Original blot image for Figure 3C – Beta-Actin for pGluA1-2

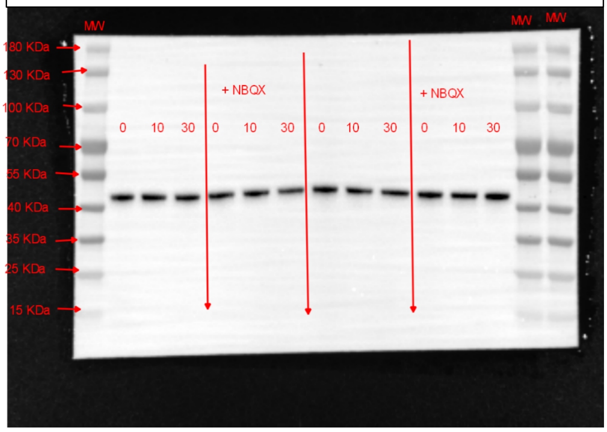

Original blot image for Figure 3C – pGluA1-3

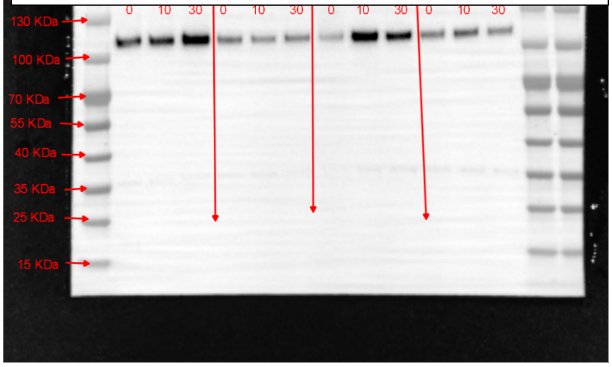

Original blot image for Figure 3C – Beta-Actin for pGluA1-3

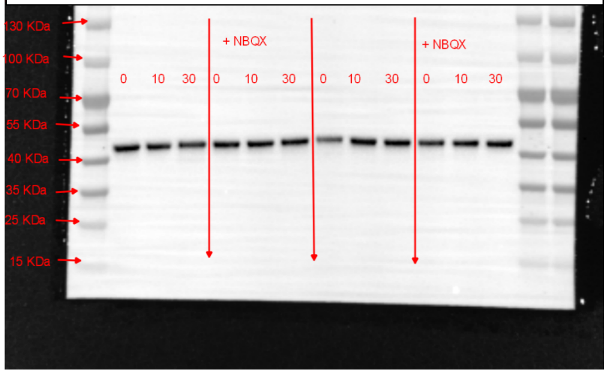

Original blot image for Figure 3C – GluA1-1

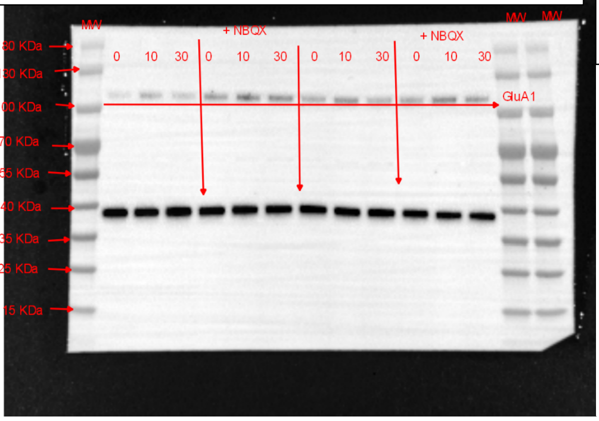

Original blot image for Figure 3C – Beta-Actin for GluA1-1

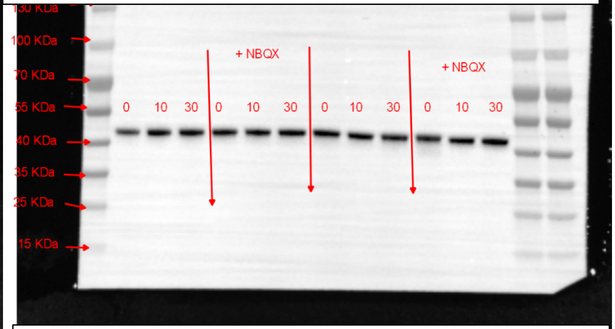

Original blot image for Figure 3C – GluA1-2

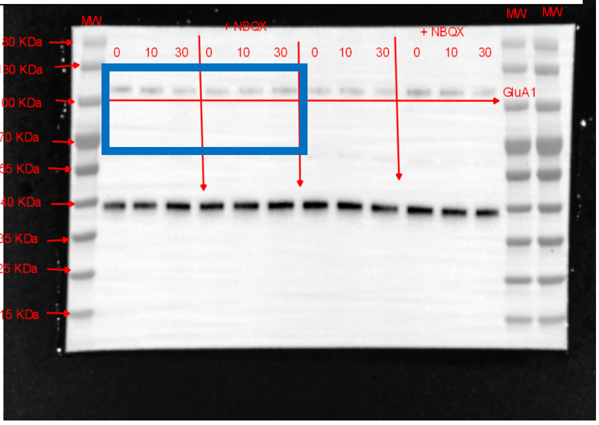

Original blot image for Figure 3C – Beta-Actin for GluA1-2

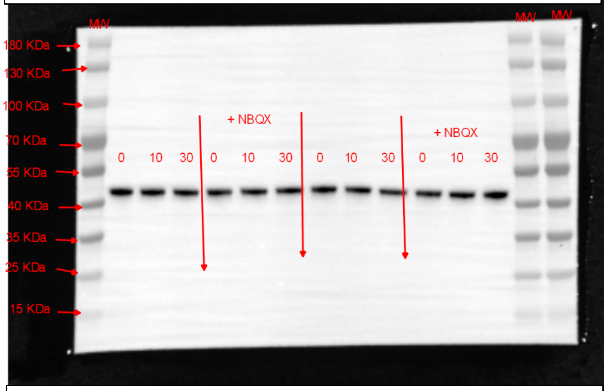

Original blot image for Figure 3C – GluA1-3

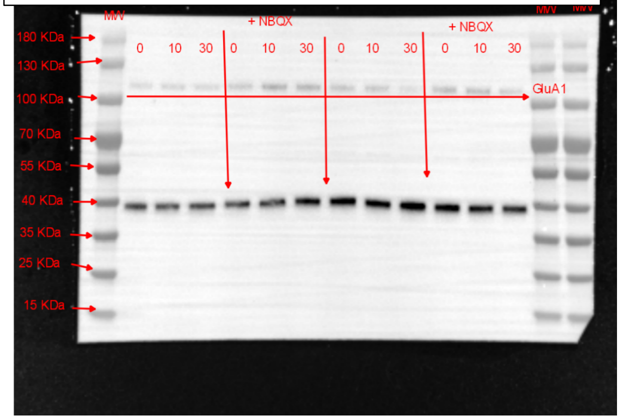

Original blot image for Figure 3C – Beta-Actin for GluA1-3

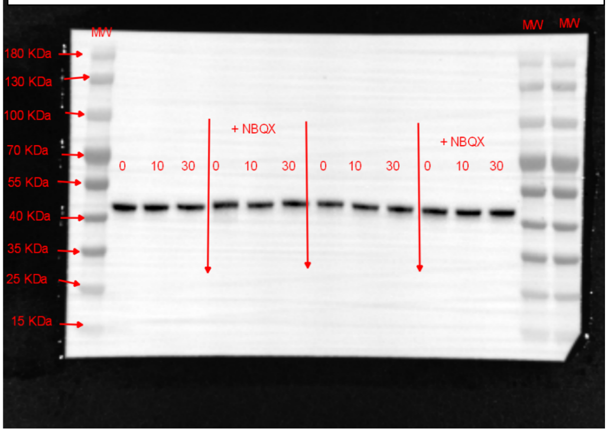

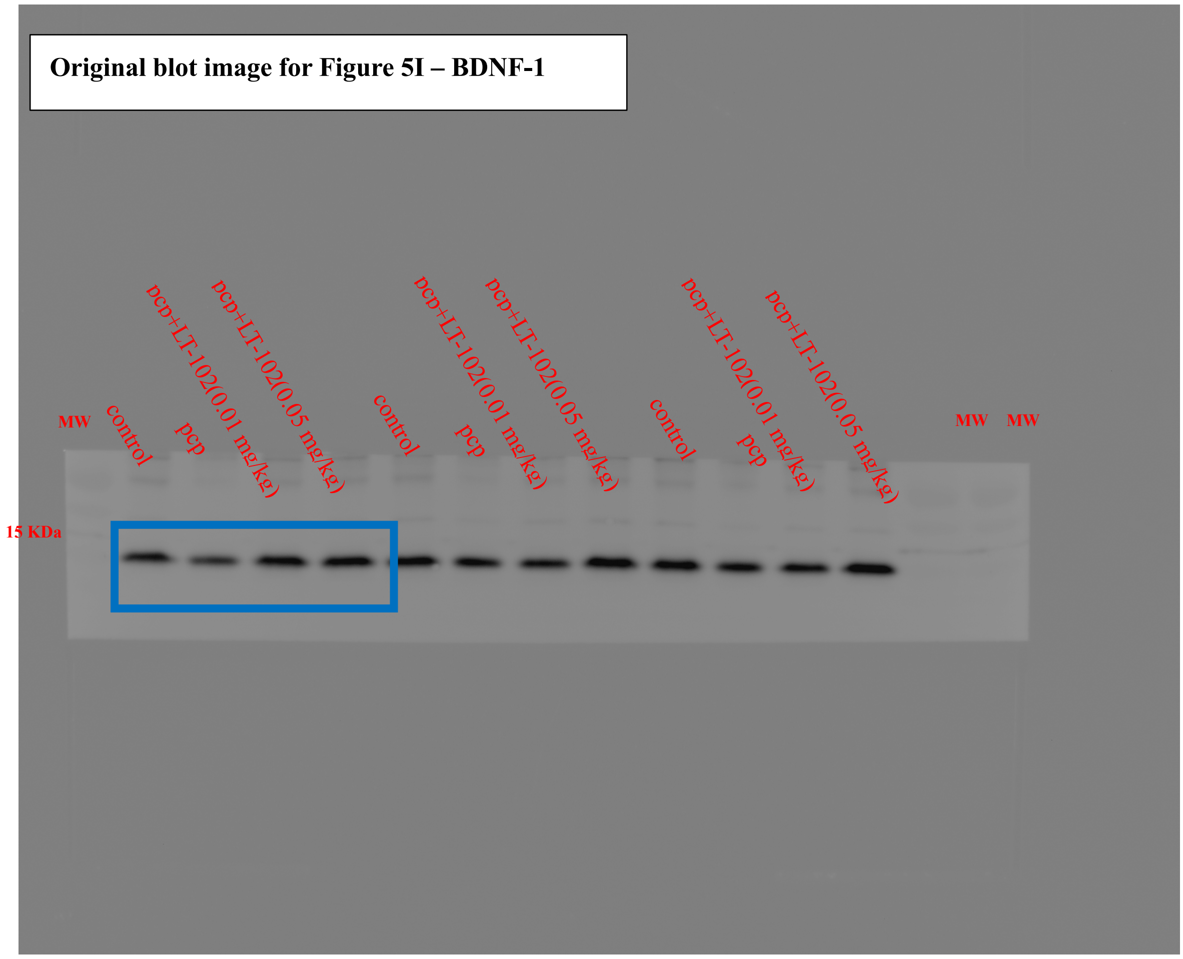
Original blot image for Figure 5I – BDNF-1
MW
control
pcp
pcp+LT-102(0.01 mg/kg)
pcp+LT-102(0.05 mg/kg)
control
pcp
pcp+LT-102(0.01 mg/kg)
pcp+LT-102(0.05 mg/kg)
control
pcp
pcp+LT-102(0.01 mg/kg)
pcp+LT-102(0.05 mg/kg)
MW
MW
15 KDa

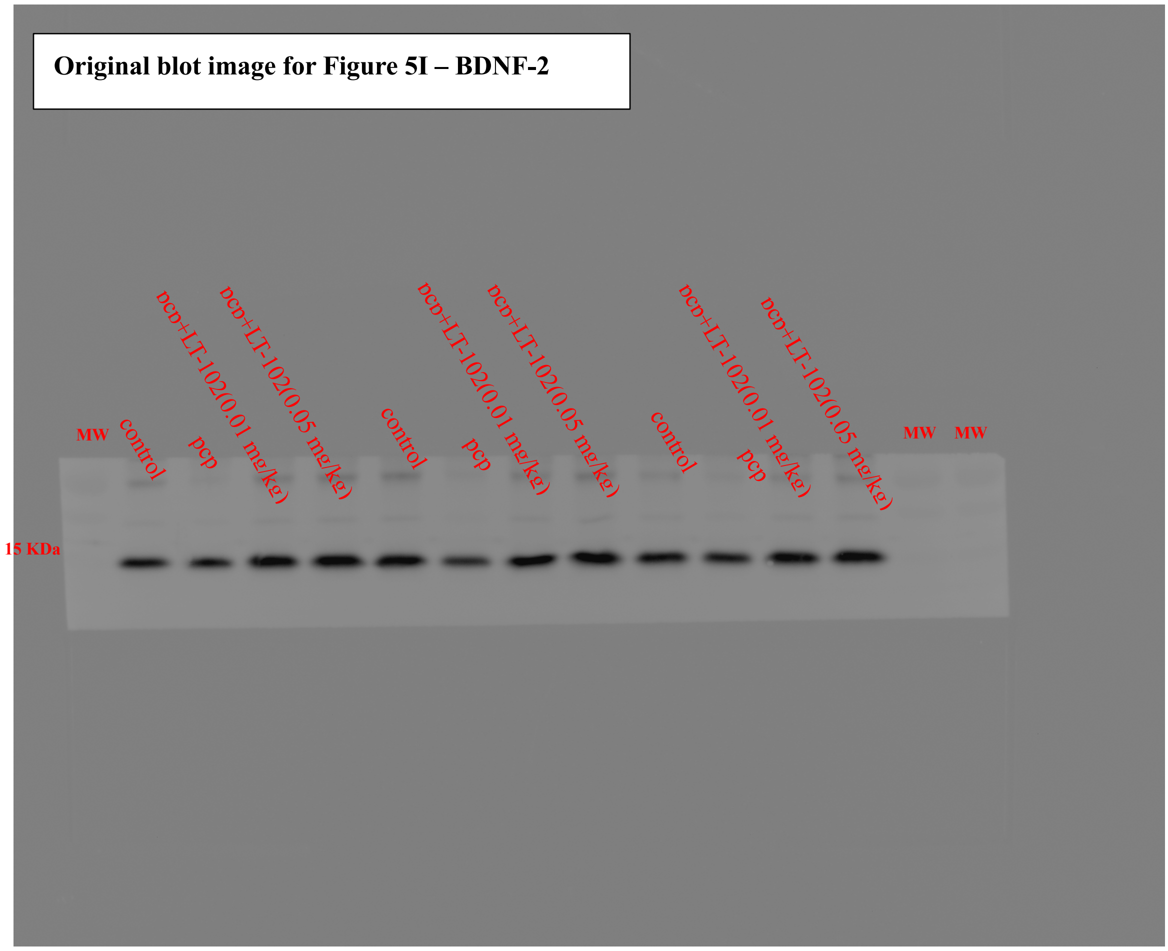
Original blot image for Figure 5I – BDNF-2
MW
control
pcp
pcp+LT-102(0.01 mg/kg)
pcp+LT-102(0.05 mg/kg)
control
pcp
pcp+LT-102(0.01 mg/kg)
pcp+LT-102(0.05 mg/kg)
control
pcp
pcp+LT-102(0.01 mg/kg)
pcp+LT-102(0.05 mg/kg)
MW
MW
15 KDa

Original blot image for Figure 5I – pGluA1-1

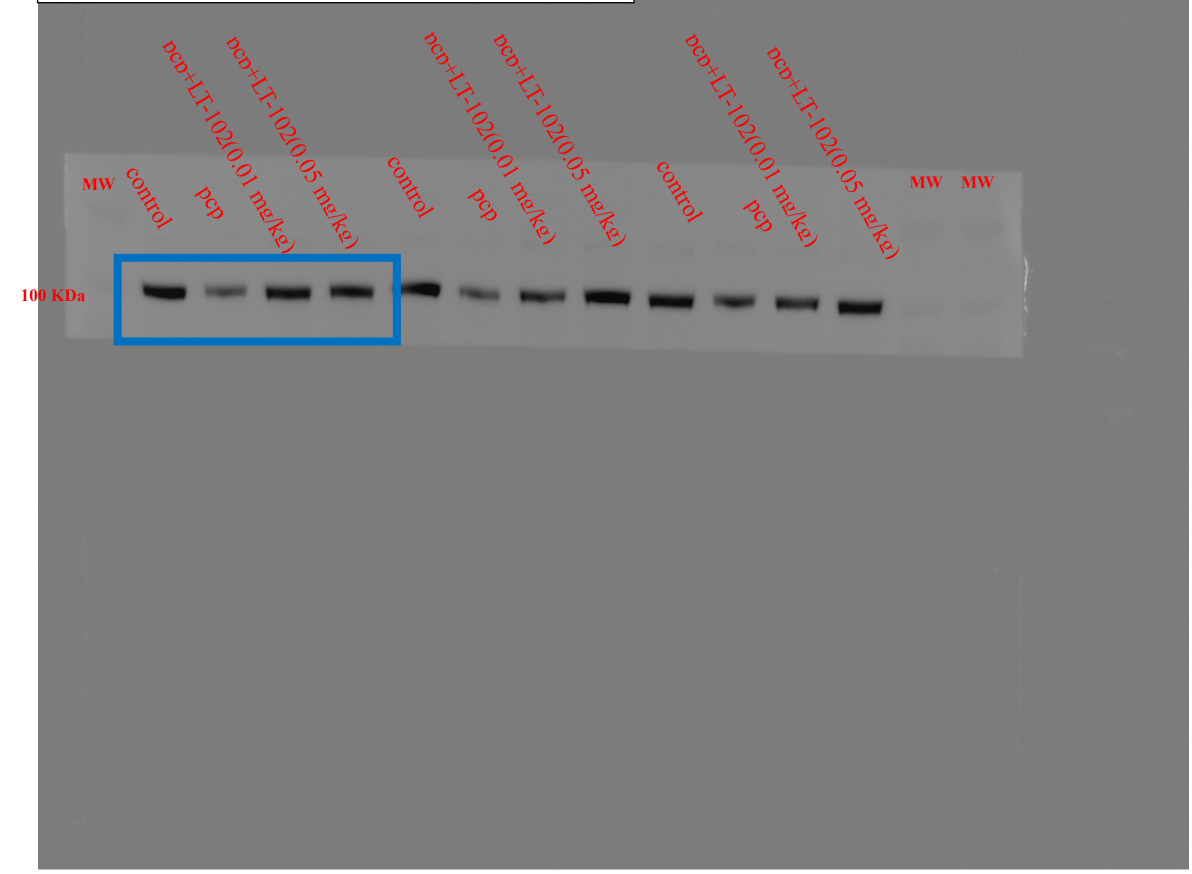

Original blot image for Figure 5I – pGluA1-2

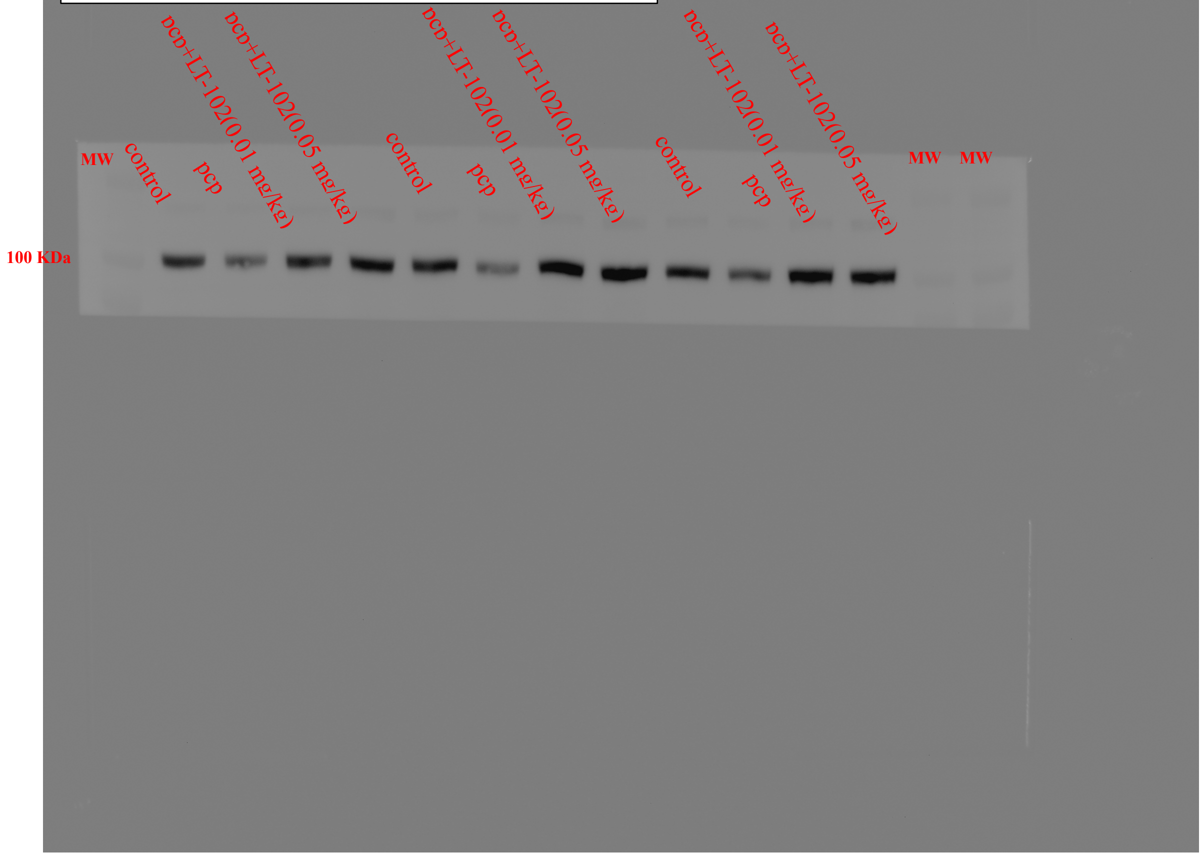

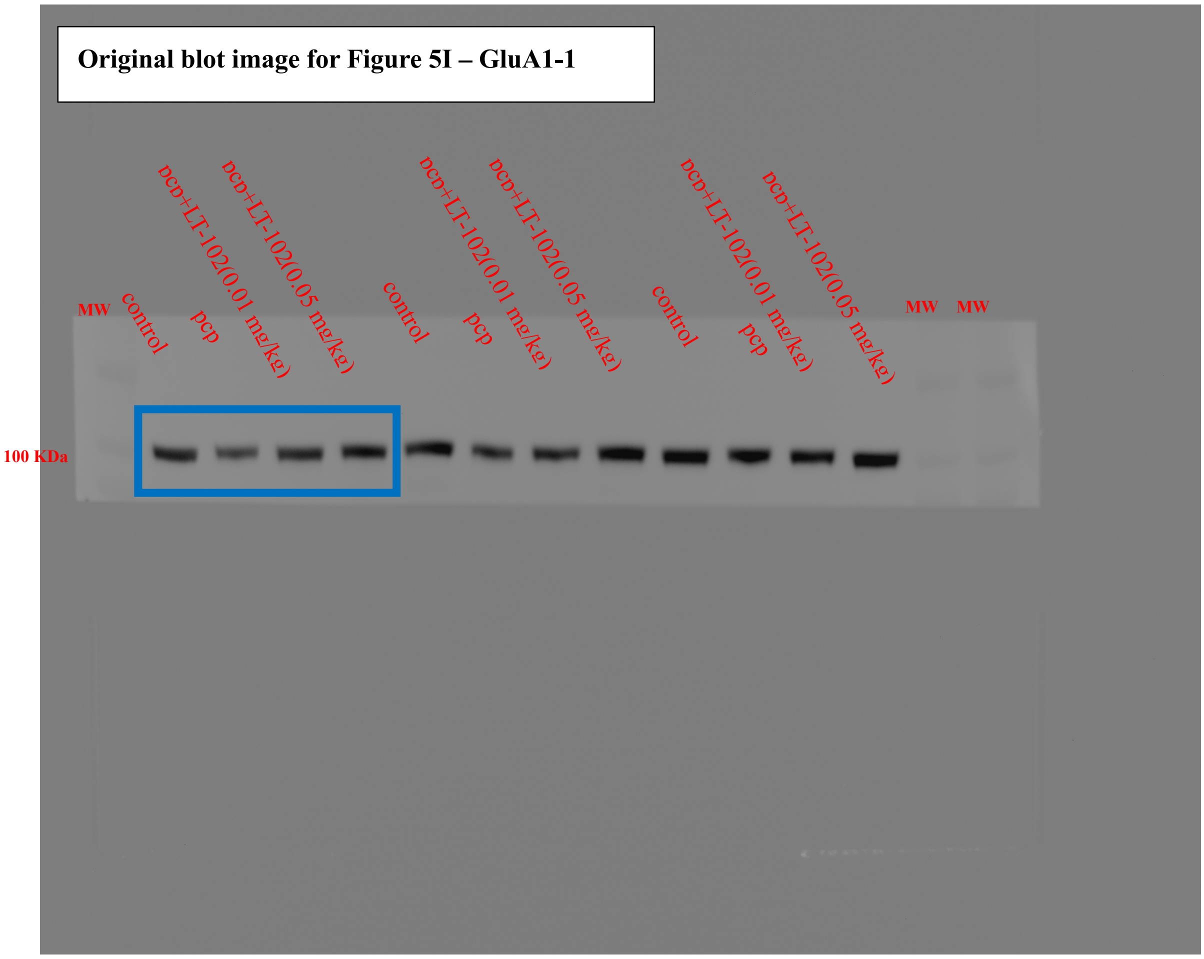

Original blot image for Figure 5I – GluA1-1

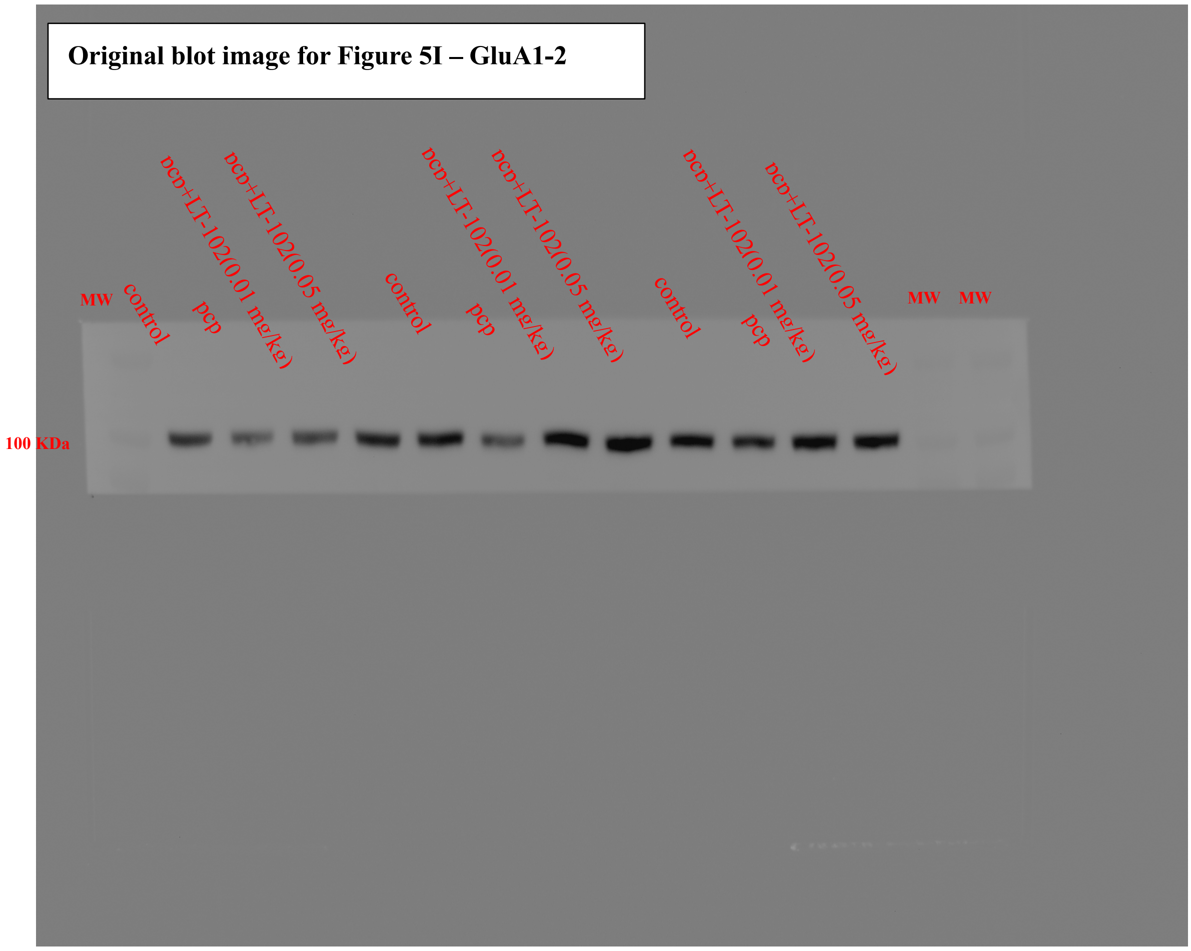

Original blot image for Figure 5I – GluA1-2

Original blot image for Figure 5I – Beta-Actin-1

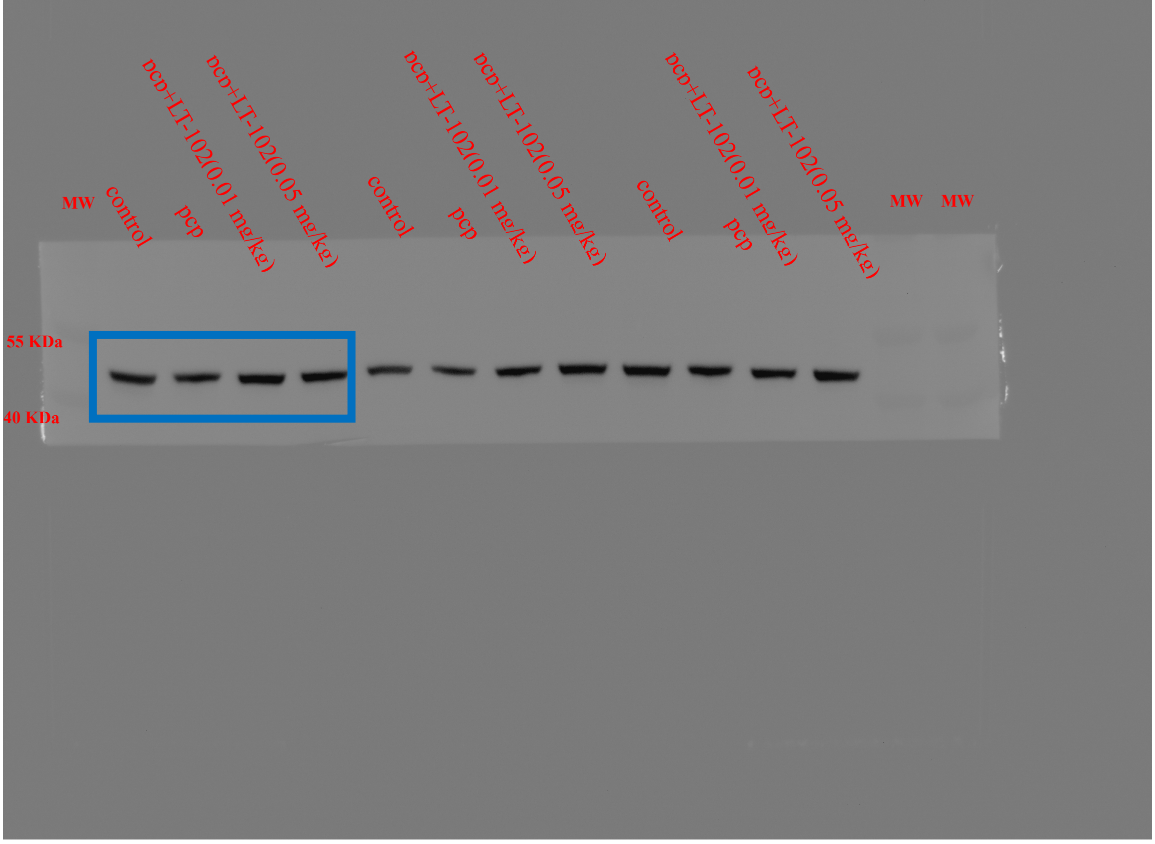

Original blot image for Figure 5I – Beta-Actin-2

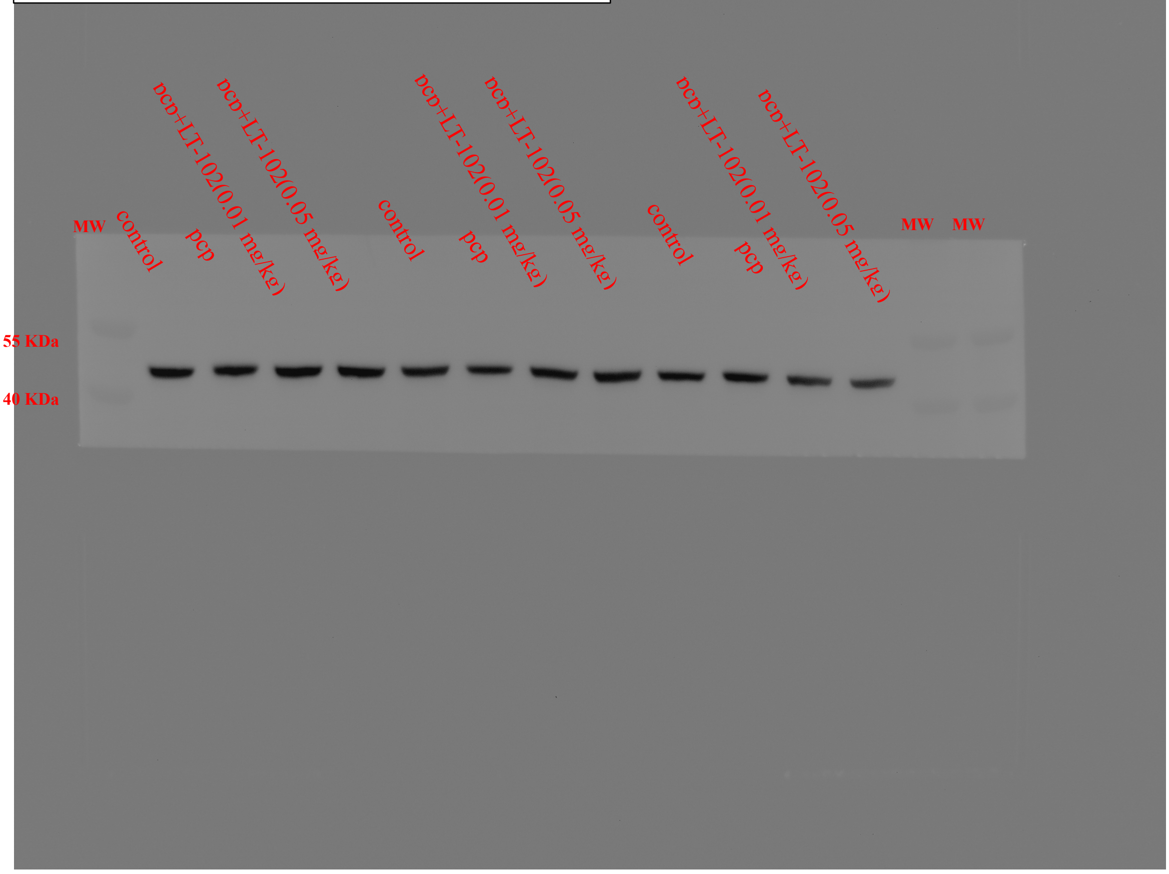

Supplement: Supplementary file 2 — Data S1. [file CNS-30-e14713-s001.pdf]
